# Genomic Designs of rAAVs Contribute to Pathological Changes in the Livers and Spleens of Mice

**DOI:** 10.1155/2022/6807904

**Published:** 2022-03-20

**Authors:** Patrick L. Mulcrone, Junping Zhang, P. Melanie Pride, Anh K. Lam, Dylan A. Frabutt, Susan M. Ball-Kell, Weidong Xiao

**Affiliations:** 1 Herman B Wells Center for Pediatric Research, Indiana University, USA; 2 Department of Pediatrics, Indiana University, USA; 3 Department of Microbiology & Immunology, Indiana University, Indianapolis, IN, USA; 4 Department of Biology, Bradley University, Peoria, IL, USA

## Abstract

Recombinant AAV (rAAV) gene therapy is being investigated as an effective therapy for several diseases including hemophilia B. Reports of liver tumor development in certain mouse models due to AAV treatment and genomic integration of the rAAV vector has raised concerns about the long-term safety and efficacy of this gene therapy. To investigate whether rAAV treatment causes cancer, we utilized two mouse models, inbred C57BL/6 and hemophilia B Balb/C mice (HemB), to test if injecting a high dose of various rAAV8 vectors containing or lacking hFIX transgene, a Poly-A sequence, or the CB or TTR promoter triggered liver fibrosis and/or cancer development over the course of the 6.5-month study. We observed no liver tumors in either mouse cohort regardless of rAAV treatment through ultrasound imaging, gross anatomical assessment at sacrifice, and histology. We did, however, detect differences in collagen deposition in C57BL/6 livers and HemB spleens of rAAV-injected mice. Pathology reports of the HemB mice revealed many pathological phenomena, including fibrosis and inflammation in the livers and spleens across different AAV-injected HemB mice. Mice from both cohorts injected with the TTR-hFIX vector demonstrated minimal adverse events. While not tumorigenic, high dose of rAAVs, especially those with incomplete genomes, can influence liver and spleen health negatively that could be problematic for cementing AAVs as a broad therapeutic option in the clinic.

## 1. Introduction

Gene therapy is a powerful biological tool being implemented in researching and treating several pathologies. Adeno-associated viral (AAV) vectors, a type of viral gene therapy with low immunogenicity and low rate of host genome integration, have been approved to treat diseases such as Leber congenital amaurosis and spinal muscular atrophy [1–3]. The clotting disorder hemophilia is also being studied with this virus-based gene therapy, as AAVs are used to correct defects in the clotting proteins factor VIII (hemophilia A) and factor IX (hemophilia B) [4]. While AAV treatments for both types of hemophilia are very close to FDA approval, questions of long-term safety and efficacy raised by the research community regarding AAVs need to be addressed.

AAV is a nonenveloped DNA virus that contains a genome as large as5 kbs. AAVs are desirable for gene therapy as they are reported to cause a relatively low immune response in vivo and require helper viruses to achieve successful infection [5–7]. For hemophilia A and B, recombinant AAVs (rAAV) containing FVIII or FIX, respectfully, are designed for delivery to the liver and induce production of the clotting factor protein that is absent [1, 4]. While translational studies and clinical trials demonstrated limited side effects from these AAV treatments, concerns about long-term effects of AAV treatments are an area of focus for the research community [7–10]. Several research groups report that in mouse models of the lysosomal storage disease mucopolysaccharidosis VII (MPSVII) and methylmalonic acidemia (MMA), mice injected with AAVs containing strong, nonspecific promoters developed hepatocellular carcinoma (HCC) at greater rates than non-AAV controls or AAVs containing more specific promoters [11–13]. Moreover, male C3H/HeJ mice subjected to liver injury that also received self-complementary (sc) AAV-CMV-eGFP or scAAV-CBA-null vector treatment developed HCC at a higher rate compared to non-AAV controls [14]. In the majority of these AAV murine studies that report HCC incidences, results also show the rare, biological phenomenon of AAV genomic integration into the host genome. This is concerning, as components of certain promoters and the ITRs of the rAAV insert into or shift the genetic sequences of oncogenes and tumor suppressors in ways that potentially contributed to HCC development [12–14]. Relating these results to the clinic, persons with blood disorders and blood-borne diseases are reported to have elevated incidences of lymphomas and liver cancers [15, 16]. Additionally, retrospective analyses of an array of 1,461 human patient HCC biopsies revealed AAV2 and AAV13 positivity in 8% of tumor tissues, highlighting questions about whether this parvovirus contributed to tumor viability and/or progression [17]. Clarifying these safety discrepancies around liver tumor development is crucial for the gene therapy field, as research on how AAVs can be used to target cancer as a therapeutic agent has increased. To date, AAVs have not been directly linked to causing any sort of disease in humans or to any cancer, and these liver pathologies are less common in large animal models of AAV therapy such as cats and monkeys [18–21]. Yet, further investigation into whether liver fibrosis and HCC are caused by AAVs is warranted.

In this study, our goal was to monitor and assess whether a high dose injection of various rAAV8 vectors caused detectable liver cancer and/or fibrosis in a healthy mouse model (C57BL/6) and a mouse model of hemophilia B (Balb/C, FIX Exon1–3 deleted) over a time course of 6.5 months, using body weight, ultrasound imaging, and postmortem histology as means of detection [22]. We used rAAVs containing either the commonly used strong promoter CB (chicken *β*-Actin) or the liver-specific promoter TTR (transthyretin), as well as the presence or absence of the transgene for factor IX, and a Poly-A sequence, a key component for RNA export and translation [23–25]. We implemented ultrasound imaging as a means of monitoring liver health during the AAV experiments and compared the assessments from that modality to postmortem analyses of the livers and spleens via H&E staining and Masson’s Trichrome histology. Our results suggest vector design influences several aspects of liver and spleen biology, and numerous methods of analysis are necessary to comprehensively detect these pathologies in AAV in vivo studies.

## 2. Materials and Methods

### 2.1. In Vivo Mouse Studies with rAAV8.

Two in vivo studies were performed, one using C57BL/6 mice, and the other with hemophilia B mice on a Balb/C background [22, 26]. Hemophilia B mice with a targeted deletion of Exons 1–3 of the murine FIX gene had been bred on the Balb/C background [22]. All animals were maintained in the laboratory animal resource center at Indiana University–Purdue University, Indianapolis (IUPUI). All animal experiments were performed as per the guidelines of Institutional Animal Care and Use Committee (IACUC). Our protocol limited us to a long-term study of 194 days post AAV injection.

8–10-week-old male mice were intravenously injected via the tail vein with 2 × 10^12^ viral genomes with 1 of 5 recombinant AAV (rAAV8) (Supplemental Fig. 1C) constructs in a volume of 200 *μ*ls. This dose approximately equates to 6 × 10^13^ vg/kg based on the average weight of 24.4 g for the mice at injection used in these studies. PBS was injected into the control mice. Prior to injection, mice were placed under a heat lamp for 10 mins to dilate vessels to ease injections and then monitored for 30 mins postinjection for any complications. After day 0 and day 3 body weight measurements, weights were taken weekly for each mouse. At sacrifice or endpoint (D194 postinjection), we measured a final body weight before euthanasia and soft tissue collection.

### 2.2. Ultrasound Imaging.

Using a Vevo 2100, ultrasound images were taken of mouse livers at D90 and D194 during the *in vivo* studies [27–30]. Mice were anesthetized using isoflurane and then placed supine to expose abdomen for imaging. Hair was removed and imaging gel applied prior to imaging livers with an MS550D probe (22–55 MHz). Both M-mode and B-mode images at a depth of 10 mm were collected; M-mode images were analyzed for echogenicity via average pixels of liver using ImageJ (http://ij.imjoy.io). Whole liver echogenicity was assessed by tracing nonvascular components of the liver image and measuring average pixels. Additionally, a region of interest near the portal triad present in all the images was assessed for differences. B-mode images were used to anatomically identify the portal triad (portal vein, hepatic artery, and bile duct). Heart rate and body temperature of all mice were continually monitored during imaging process.

### 2.3. Histology, Microscopy, and Pathology.

The liver, spleen, heart, and lungs were collected, fixed in 10% neutral buffered formalin for 48 hrs at 4C, and then stored in 70% ethanol at 4C prior to histological processing. Liver weights and spleen weights were collected at sacrifice. Mouse livers and spleens were processed in paraffin for histological analysis. Processed tissues were embedded in paraffin blocks and cut into 5-micron sections. Glass slides were stained with hematoxylin and eosin (H&E) prior to histopathologic examination. H&E sections were analyzed on an Olympus BX41 microscope fitted with a DP71 camera at magnifications of 10, 20, 40, and 60x. Liver and spleen fibrosis was scored as per Kleiner et al. and the American Veterinary Medical Association [31–35]. Masson’s Trichrome staining and F4/80 immunohistochemistry were performed per protocols at the IUSM Immunohistochemistry Core. Trichrome stains were imaged on an Olympus IX73 inverted microscope at 4x, 10x, and 20x objectives. Percent of collagen area (blue color) of the livers and spleens was assessed via MetaMorph software at the Indiana Center for Biological Microscopy [36]. F4/80 stains were also imaged on the same microscope at 10x and 20x objectives.

### 2.4. AAV Construct Design, rAAV Production, and Titer Determination.

The parent plasmids pDS-AAV-CB-EGFP, pDS-AAV-CB-hFIX (co), and pDS-AAV-TTR-hFIX (co) were in stock in our lab [37]. pDS-AAV-CB-PolyA was constructed by removing a 994 bp fragment from pDS-AAV-CB-eGFP using AccI and SacII (New England Biolabs) digestion; pDS-AAV-CB was constructed by removing a 1210 bp fragment from pDS-AAV-CB-EGFP using ClaI and SacII (New England Biolabs) digestion; pDS-AAV-TTR-PolyA was constructed by removing a 1413 bp fragment from pDS-AAV-TTR-FIX (co) using BamHI and SacI digestion. Subsequently, the above various fragments were filled-in by T4 DNA polymerase and then self-ligated. The constructs were further confirmed by digital droplet PCR (Bio-Rad).

A triple plasmid cotransfection method was used to produce rAAVs as described previously [38, 39]. One part vector plasmid (Supplemental Fig. 1A, 1B), one-partAAV8 helper plasmid, and one-part miniadenovirus function helper plasmid pF*Δ*6 were cotransfected into HEK293 cells cultured in roller bottles at a ratio of 1 : 1 : 1. The transfected cells were harvested 4 days later. rAAVs were then purified by two rounds of cesium chloridegradient ultracentrifuge. After extensive buffer exchange against phosphate-buffered saline with 5% d-sorbitol, the peak fractions of purified virus were pooled and stored at −80°C before administration.

Genome titers were determined by qPCR assay following the previous protocol [39]. Briefly, 10 *μ*ls of purified virus was treated in 90 *μ*ls DNase I buffer (DNase I, 1 U) at 37°C for one hour and then heated at 85°C for 20 min to inactivate DNase I. Next, 50 *μ*ls lysis buffer (direct qPCR lysis buffer) containing 0.5 mg/ml proteinase K was added, incubated for 1 hour at 56°C and heated at 95°C for 15 min. rAAV genomes were amplified using various primers, provided in Supplemental Table 1, and the titer for each was calculated. SnapGene software was used to document all vector processes, production of new vectors, and storage of plasmid maps and enzyme information.

### 2.5. Activate Partial Thromboplastin Time (APTT).

We collected blood from the mice by retroorbital bleeding on day 90 and at the endpoint, day 194 postinjection. After anesthesia using isoflurane gas, nonheparinized microhematocrit tubes (Fisherbrand #22–362-574) were applied to the back of the eye with pressure, causing slight rupture and blood flow. Two tubes of blood were collected per mouse placed into a 1.5 ml tube containing 20 *μ*ls of 3.8% sodium citrate on ice, forming a solution of approximately 9 : 1 ratio of blood to sodium citrate. Mice were monitored for 15 mins after blood collection for any complications. Samples were spun for 15–20 minutes at a speed of 15,000 rpm at 4°C. We collected the plasma after the centrifugation and performed 1-step APTT using Stago Diagnostica equipment [40]. Briefly, a 1 : 1 : 1 mixture of Stago APTT buffer, murine plasma, and factor IX (FIX)-deficient plasma were incubated for 170 s in Stago apparatus in the presence of a moving bead. Then, 50*μ*ls of calcium chloride (CaCl_2_) was added to induce clot formation. Times were recorded, indicating when the bead stopped moving due to clot formation. Clot time is presented as time relative to 1 unit based on standards using serial dilutions of FIX-containing plasma.

### 2.6. Statistics.

GraphPad Prism 9.1.2 software was used to calculate all statistics. Unless otherwise stated, data are presented as means ± the standard error mean (SEM). For experiments comparing more than 2 groups, one-way ANOVA was used with Dunnett’s post hoc test unless stated differently in a specific figure legend. A simple linear regression analysis was used for all correlation data in this manuscript. For all tests, a *p value* less that 0.05 was considered significant. For survival data, a Mantel-Cox Log-rank test was used.

## 3. Results

### 3.1. Vector Design for In Vivo Studies.

To design our AAV vectors, we used enzymatic digest to remove specific components of the plasmid genome of pDS-AAV-TTR-hFIX and pDS-AAV-CB-eGFP and purified the resulting products for *in vivo* injection [39]. The FIX-containing vectors (pDS-AAV-CB-hFIXco and pDS-AAV-TTR-hFIXco) are in-house vectors already produced by our lab. The pDS-AAV-TTR-hFIX and pDS-AAV-CB-eGFP vectors were used as starting products to produce the following vectors: pDS-AAV-CB, pDS-AAV-CB-PolyA, and pDS-AAV-TTR-PolyA. Supplemental Fig.1A shows the gel simulation from SnapGene software that contains the sequence map information for the plasmids used in this study. Supplemental Fig.1B is the actual gel, with the bands identified for excision and purification by the blue arrows. Supplemental Fig.1C is graphical representations of the final AAV product that were injected *in vivo*. These 5 vectors were injected into two different sets of mice, a C57 BL/6 mouse model, and a hemophilia B mouse model on a Balb/C background.

### 3.2. Mouse Weights Do Not Differ in C57BL/6 In Vivo Study.

**Figure 1:**
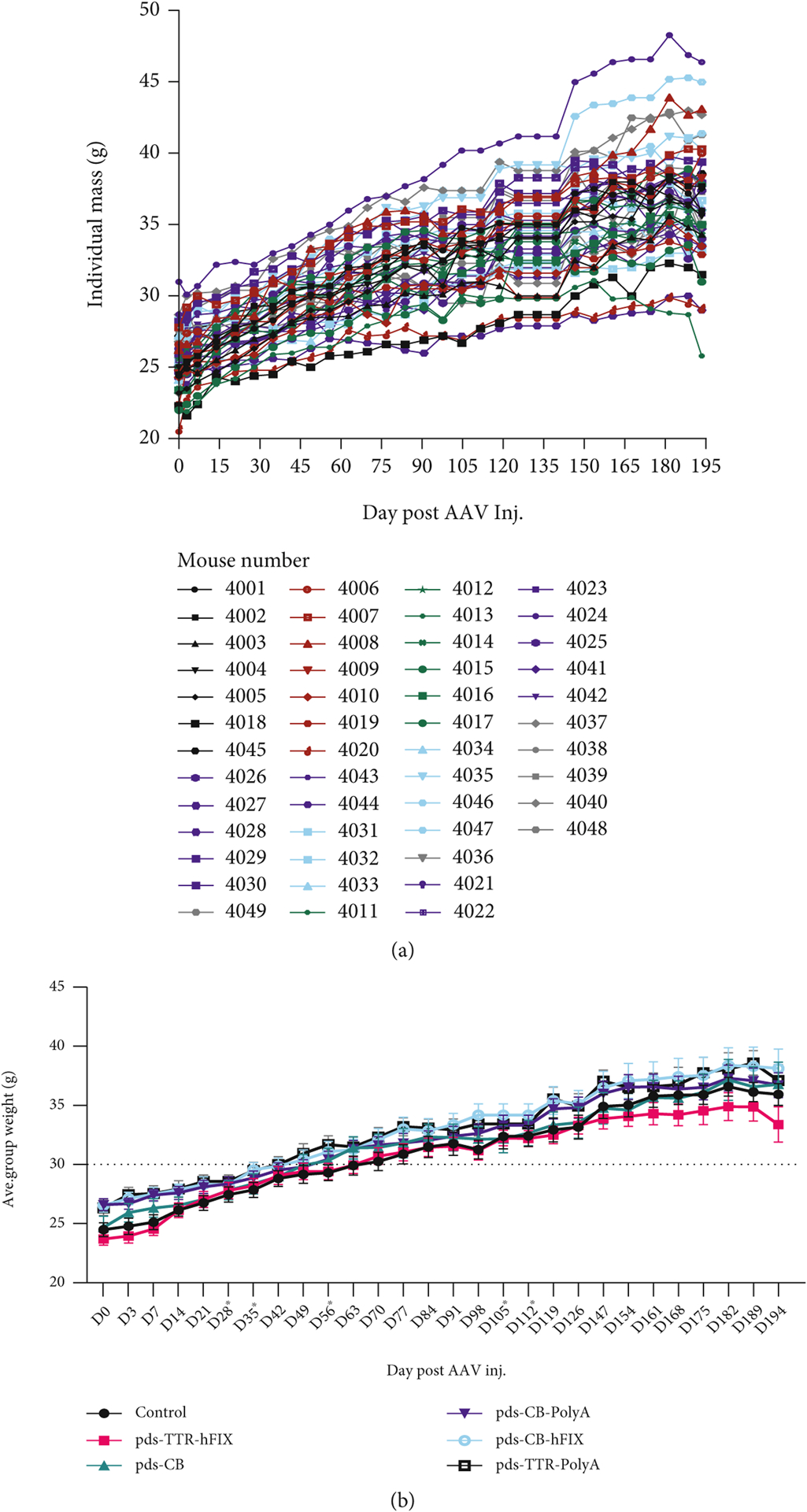
C57BL/6 mice have similar weights regardless of AAV injection. (a) Individual weights across experiment. (b) Weights grouped by injected vector. No significant differences in weights found at endpoint (D194) postinjection.

As the main goal of our study is to determine the safety of our AAV8 constructs *in vivo*, we used the common inbred C57BL/6 mouse model. We measured mouse weights weekly throughout the study. A common side effect of cancer is cachexia, which includes dramatic weight loss and muscle wasting [41]. Therefore, if a severe side effect of the vectors occurred, mouse weights would drop. Individual C57BL/6 mouse weights ranged from low 20 s to low 30 s g at D0 and continued to steadily climb throughout the study (Figure 1(a)). Some of the mice’s weights dropped in the final two weeks, but not severe enough to suggest the presence of a liver tumor. When the individual weights were grouped together, all groups exhibited a similar pattern of weight change, and no statistical difference was detected (Figure 1(b)).

### 3.3. Ultrasound Reveals Differences in Liver Echogenicity in C57BL/6 Injected with rAAVs.

**Figure 2:**
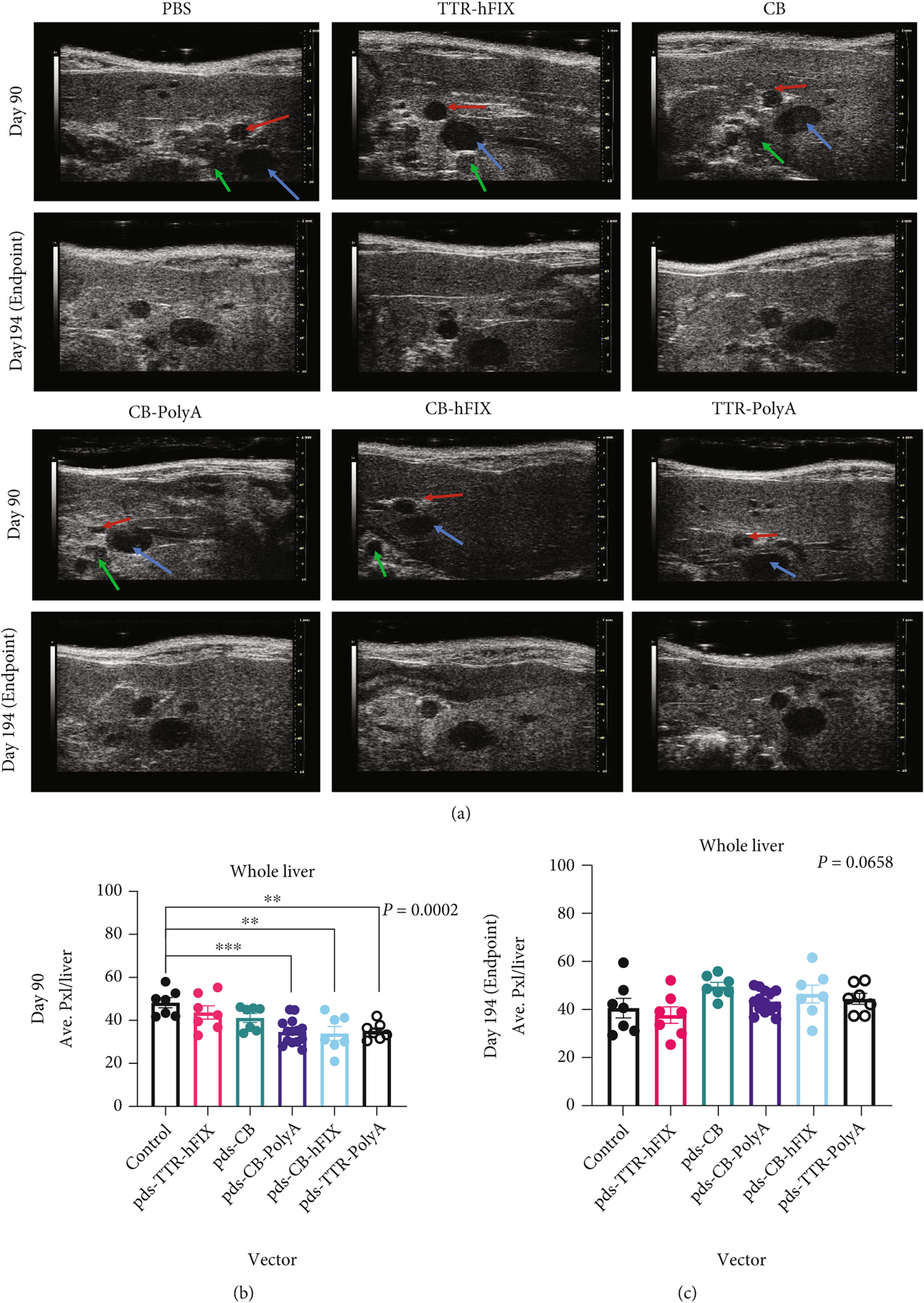

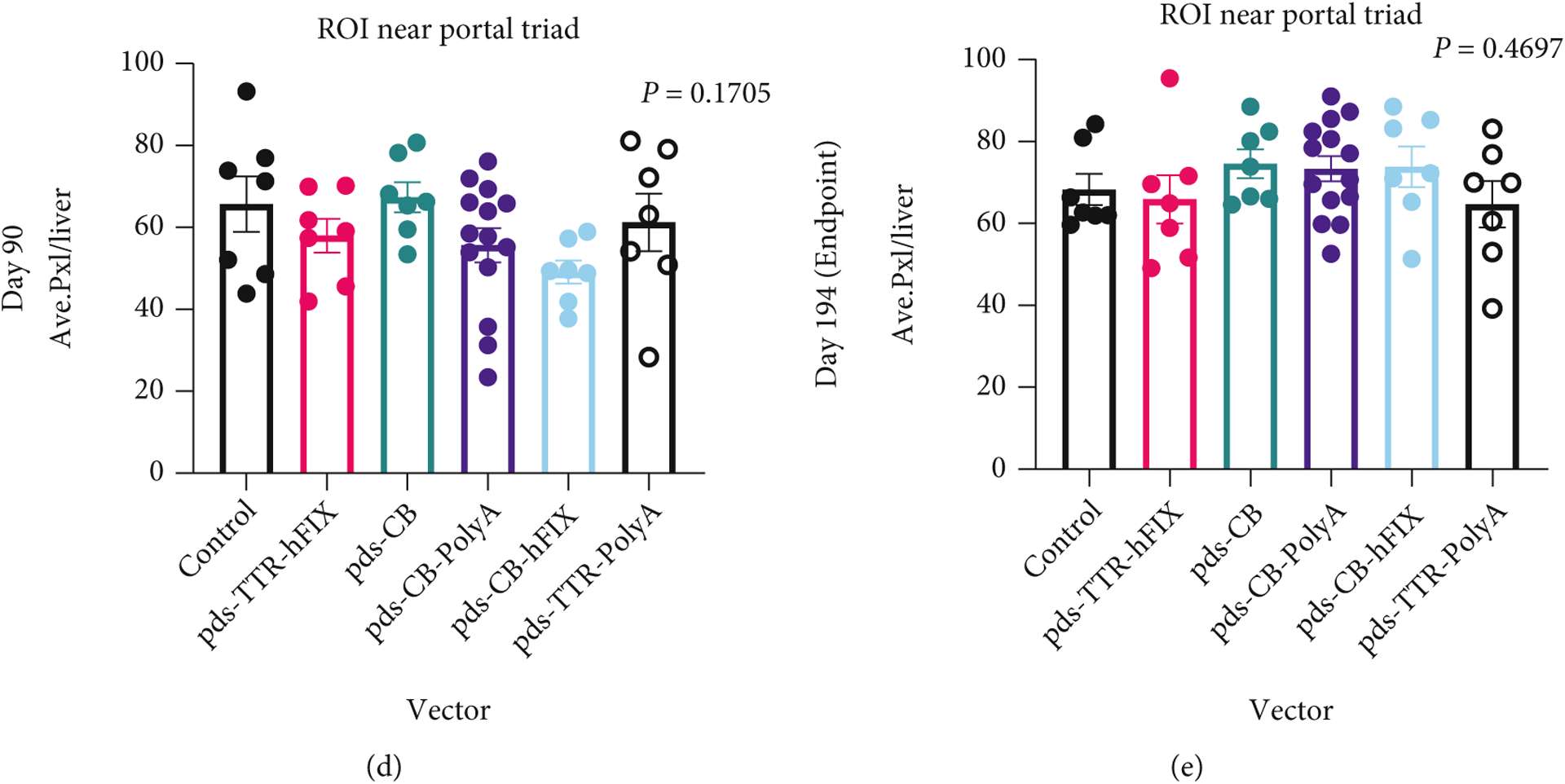
Echogenicity measurement of all C57BL/6 mice injected with AAV. (a) Representative day 90 and day 194 liver ultrasound images for all groups of C57BL/6 mice. Arrows indicate Portal Triad features on D90 images: blue: portal vein; red: hepatic artery; and green: bile duct. (b, c) Whole-liver echogenicity analysis via average pixels of liver images. (d, e) Echogenicity analysis of a region of interest near the Portal Triad. ANOVA *p* value significant for (b) only. *p* values for (c–e) are 0.1705, 0.0658, and 0.4697. *N* = 7 for all groups except CB-PolyA (*N* = 14).

In order to assess the potential effects of the specific AAV8 constructs injected into the C57s on HCC and liver fibrosis development, we implemented ultrasound imaging of mouse livers at 2 timepoints during the experiment. M-mode images were collected at day 90 and endpoint (day 194) and analyzed for echogenicity, which manifests as white sections of the M-mode image. Representative images of each group and the two timepoints are shown in Figure 2(a). Pixel readouts of the ultrasound images demonstrate that whole liver analysis of control mice showed the highest echogenicity at day 90 (Figure 2(b)). pDS-TTR-hFIX- injected mice had a similar pixel count as controls, and the other four groups had an even lower pixel count. This pattern, however, changes at endpoint; mice injected with pDS-AAV-CB and pDS-CB-hFIX have the highest pixel count at endpoint, and control and pDS-TTR-hFIX mice have the lowest pixel count (Figure 2(c)); these differences trended toward significance. This suggests that different promoter and different components of AAV vectors could be affecting overall health of livers over time via changes in tissue composition. A region of interest near the portal triad that was consistently bright in the ultrasound images (see Materials and Methods) exhibited no significant differences among the groups at either timepoint (Figures 2(d) and 2(e)). This highlights how ultrasound imaging can be used to monitor liver health of mice with AAV treatment and the importance of performing whole-organ assessments and analyzing multiple areas of tissues to determine a true effect of AAV treatment.

### 3.4. Liver and Spleen Weights of rAAV-Injected Mice Trend Heavier in the C57BL/6 Model.

**Figure 3:**
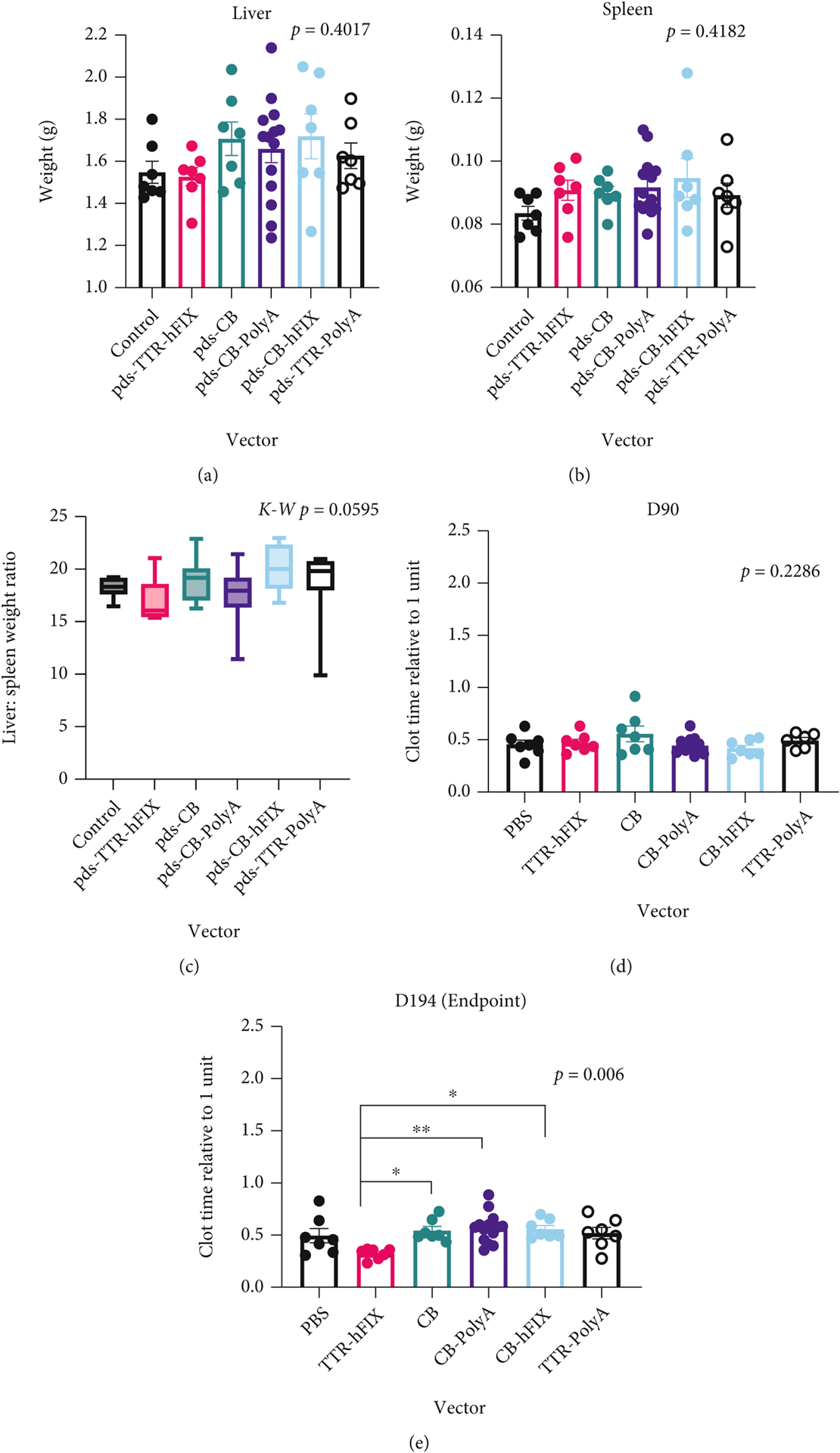
Liver weights vary while spleen weights trend heavier in rAAV-injected C57BL/6 Mice. (a) Endpoint liver weights of C57 mice. ANOVA *p* = 0.4017. (b) Endpoint spleen weights. ANOVA *p* = 0.4182. *N* = 7 for all groups except CB-PolyA (*N* = 14). (c) Liver : spleen weight ratios of C57BL/6 mice injected with rAAVs. Ratios reflect variability of the organs’ weights but are all similar. Kruskal-Wallis *p* = 0.0595. (d, e) Clot times via APTT of C57BL/6 mice injected with various rAAVs at day 90 and 194 postinjection. *N* = 14 for CB-PolyA, *N* = 7 for all other groups. ANOVA tests performed for (d) and (e).

Changes in organ weight, tissue composition, and cellular distribution are indicators of pathology and responses to challenges [31]. At sacrifice, we weighed mouse livers and spleens to determine if any gross details of these organs differed based on AAV8 injection. Whole livers of C57 control mice weighed the least of all groups, and pDS-TTR-hFIX-injected mice exhibited a similar weight to controls. Interestingly, the other four AAV groups (pDS-CB, pDS-CB-PolyA, pDS-CB-hFIX, and pDS-TTR-PolyA) had heavier liver weights (Figure 3(a)). This pattern matches that observed in the endpoint whole liver echogenicity analysis, suggesting a possible change in liver composition that manifested in a heavier organ at endpoint.

It is documented that AAV vectors can cause immune responses over time, which is a very active area of gene therapy research, as strong immune responses to AAV therapy can diminish the effects of the treatment and cause negative outcomes for patients [1, 8, 10, 42–47]. To understand if AAV injection could have altered the spleens in the C57 mice, we collected this key organ for the immune system from each mouse at endpoint and measured the weights before processing them for histological analysis. Generally, AAV-injected mice had slightly heavier spleens compared to controls. Among the AAV-injected groups, the spleen weights were similar and showed no significant difference (Figure 3(b)). This result suggests that the rAAV8 challenge did not cause an adverse change in spleen weight in the C57 mice. In order to relate the livers and spleens of each mouse to each other, the liver to spleen weight ratios of each mouse were calculated, revealing similar values among the six groups and no significant differences (Figure 3(c)). Of note, liver weights, but not spleen weights, positively correlated with endpoint whole body weight (Supplemental Fig. 2A, 2B). To test the activity of the rAAV8 vectors, activated partial thromboplastin time (APTT) assays using plasma collected fromC57BL/6 mice plasma was run; a reduction in clot time was observed in the pDS-TTR-hFIX C57BL/6 group at endpoint, demonstrating that the rAAV8 vectors were functioning properly in our animal study (Figures 3(d) and 3(e)).

### 3.5. Histological Analyses Reveal Collagen and Cellular Composition Differences in Livers and Cellular Differences in Spleens of C57BL/6 Mice Injected with CB-Containing rAAV8s.

**Figure 4:**
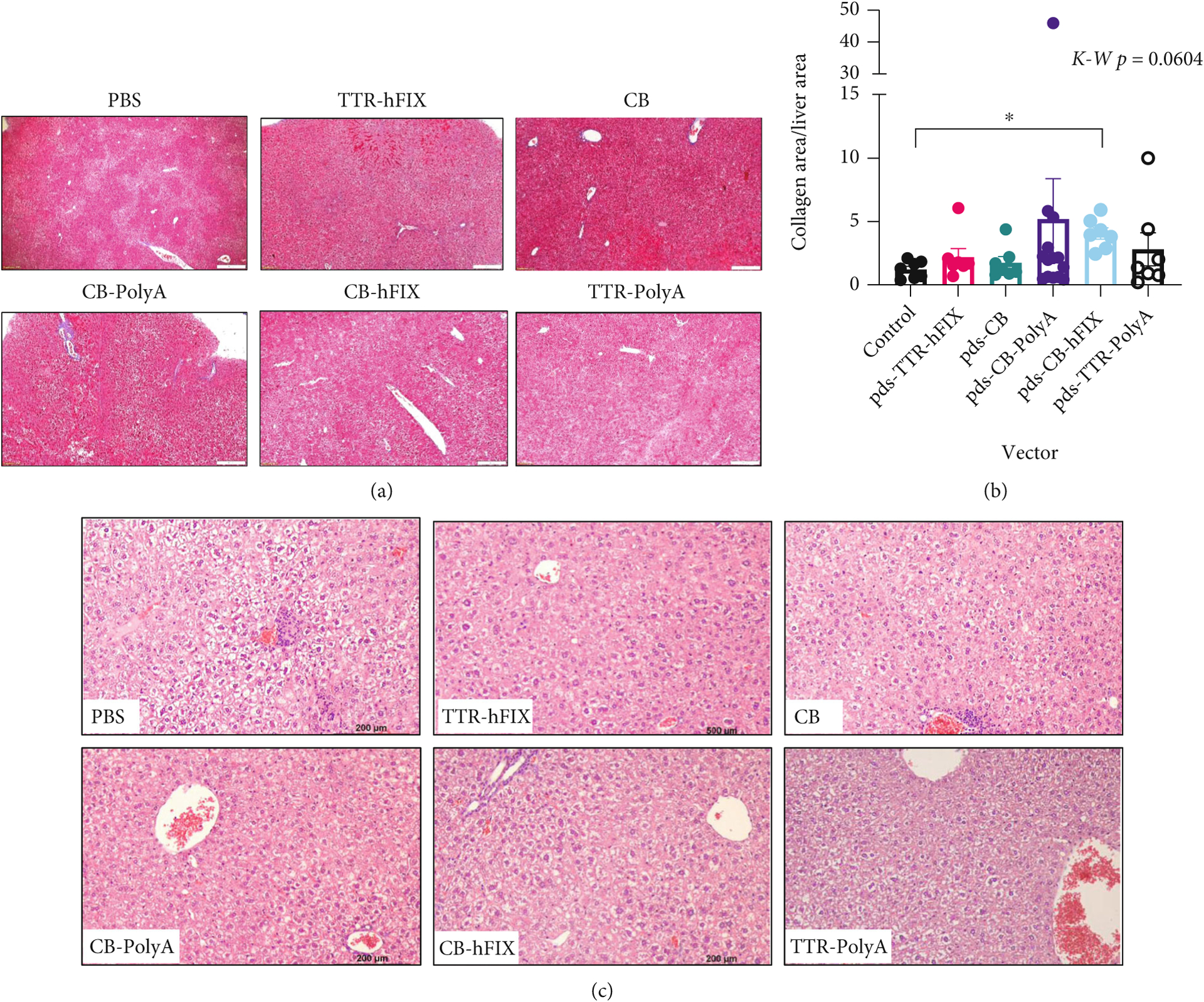
Histology of livers reveals alterations in collagenous area of AAV-injected C57s. (a) Representative 10x images of collagenous area in C57 livers. (b) Collagen area low in livers of TTR-hFIX, and high in CB-PolyA and CB-hFIX-injected mice. Kruskal-Wallis test performed. (c) H&E 20x images of C57 livers. Cytoplasmic clearing and EMH detected in several mice. *N* = 14 for CB-PolyA, *N* = 7 for all other groups.

To better understand the pathological effects of our rAAVs, we supplemented weights and ultrasound imaging analyses with histopathological analysis, which provides more comprehensive assessment of tissue composition, cellular distribution, and cellular health of the livers and spleens. Fibrosis is a concern, as this is the result of active tissue repair and increased activity of fibroblasts. This biological phenomenon can manifest as increased collagen deposition and affect organ function, with the possibility of cancer development [28, 48, 49]. Therefore, we performed Masson’s Trichrome staining to assess the levels of collagen in the livers and spleens of the C57 mice. We observed elevated collagen area in livers of pDS-CB-PolyA- and pDS-CB-hFIX-injected mouse groups compared to the control group in the C57 mice (Figures 4(a) and 4(b)). Mice injected with the pDS-TTR-hFIX vector, an rAAV8 that restores FIX levels in hemophilia B mouse models, and pDS-CB had a similar collagen deposition area compared to the controls, suggesting a low possibility of fibrosis induction in the liver. pDS-TTR-PolyA-injected mice had a slightly elevated collagen area compared to the controls. When analyzing the Trichrome stains of the spleens, no differences were observed among the groups, although four mice (2 controls, 2 pDS-CB) did have elevated collagen deposition in the spleens (Supplemental Fig. 3A, 3B). H&E staining of the C57BL/6 mice livers revealed both extramedullary hematopoiesis (EMH), which can be common in livers of mice, and cytoplasmic clearing, a reversible phenomenon connected to liver injury, in several samples (Figure 4(c)). Interestingly, no fibrosis or inflammation was observed in these livers, In the spleens, several mice across 4 groups had detectable hemosiderin, iron deposits caused by erythrocyte injury, and dysregulated iron metabolism (Supplemental Fig. 3C) [50]. The H&E staining we performed also showed no presence of HCC or tumors in the spleens, supporting the ultrasound and Trichrome staining data and reinforcing the safety of these AAVs regarding HCC development *in vivo*. Overall, these results demonstrate that C57BL/6 mice experienced minimal negative pathological changes in response to our selected AAVs and did not present with HCC 6.5 months postinjection. However, the promoter type and design of our selected vectors might play a role in altering collagen deposition and cellular iron metabolism in the livers and spleens.

### 3.6. Mouse Weights Differ in Hemophilia B In Vivo Study.

**Figure 5:**
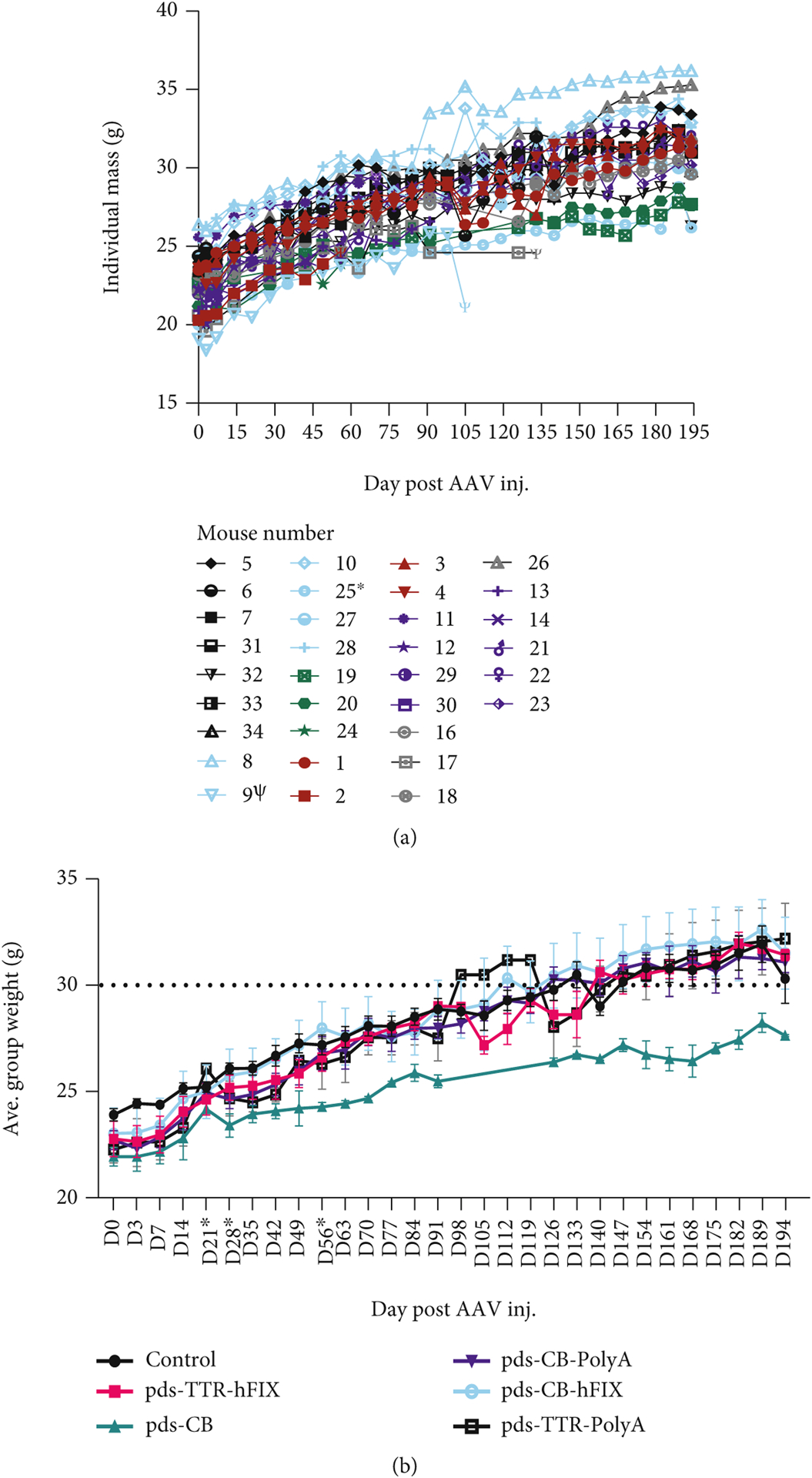
HemB mice injected with CB vector have lower weight compared to other groups. (a) Individual weights across experiment. (b) Weights grouped by injected vector. CB mice were significantly lighter at D194 postinjection.

Along with the C57BL/6 mouse model, we conducted our safety and efficacy *in vivo* study and assessments using the Balb/C hemophilia B mouse model [22, 44]. AAV gene therapy is near clinical approval for hemophilia, a blood disorder in which clotting proteins factor VIII (hemophilia A) and factor IX (hemophilia B) do not function properly, resulting in excessive bleeding after injury and abnormal clotting [1]. Therefore, concerns of long-term safety and efficacy of rAAV design and vector components is a key in improving gene therapy treatment for hemophilic patients. Hemophilia B mice were weighed weekly throughout the study; many of the mice exhibited a steady increase in weight over time (Figures 5(a) and 5(b)). pDS-CB mice had lower body weight during most of the study, while the other 5 groups had comparable weights. One-third of the mice, however, died or were euthanized prior to endpoint based on animal health and our IACUC protocol. However, these pre endpoint deaths did not lead to any survival differences among the groups (Supplemental Fig. 4).

### 3.7. HemB Mice Injected with rAAV8s Containing Incomplete Genomes Have Lower Liver : Spleen Weight Ratios and Increased Clotting Time.

**Figure 6:**
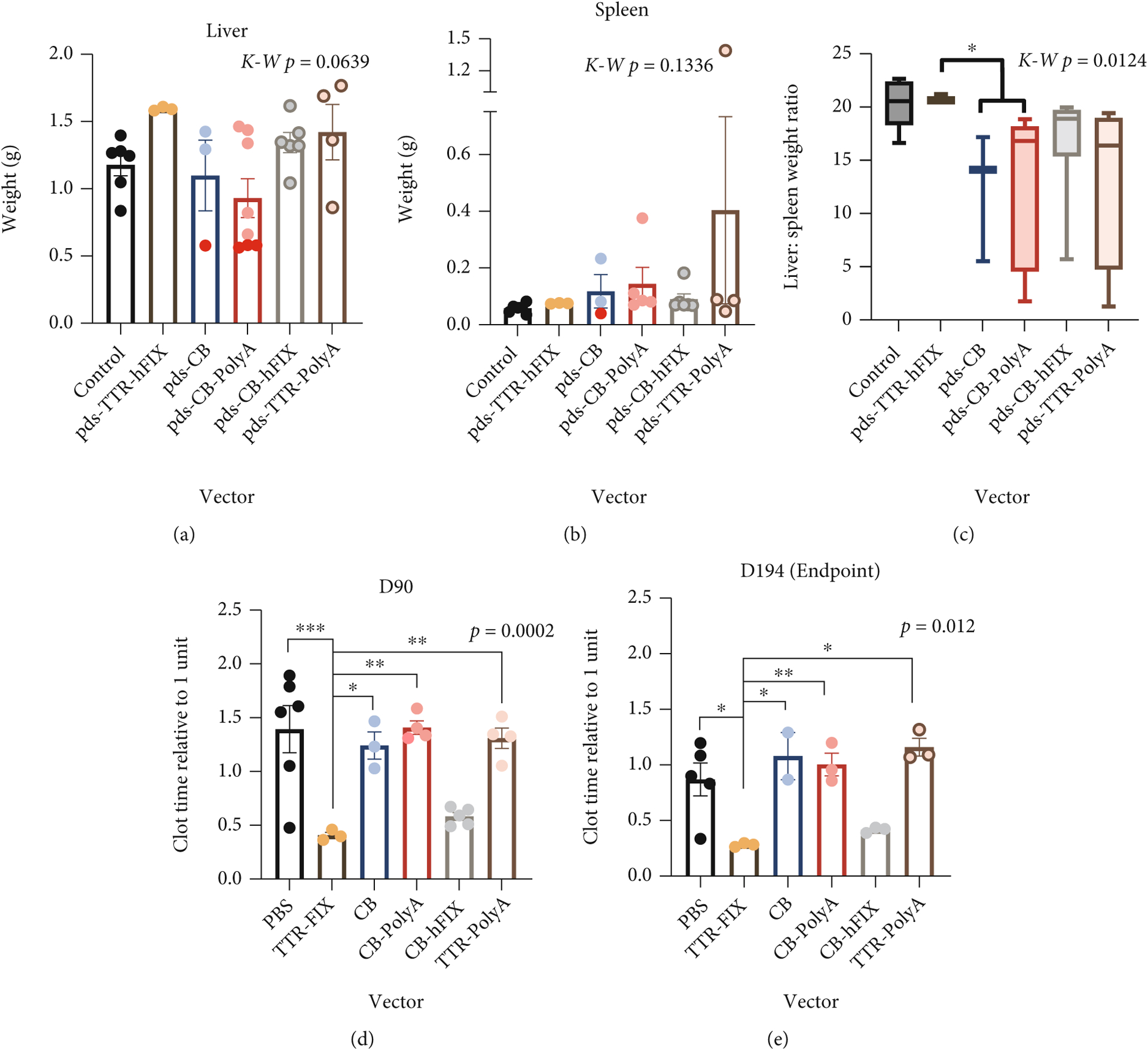
HemB mice injected with rAAVs containing incomplete genomes exhibit reduced liver: spleen weight ratios and greater clot time compared to TTR-hFIX-injected mice. (a) Liver weight mice trended towards significantly different. Kruskal-Wallis test performed, *N* = 3–8. (b) Spleen weights reveal some mice with very heavy spleens. TTR-hFIX had lowest average spleen weight of vector-injected groups. Red dots: moribund mice, Kruskal-Wallis test performed, *N* = 3–5. (c) Liver to spleen weight ratios of HemB mice injected with rAAVs. TTR-hFIX mice and controls are higher than other four groups. CB and CB-PolyA ratios are significantly lower compared to TTR-hFIX. (d) Clot time analysis of HemB mice via APTT at D90. *N* = 3–6. (e) Endpoint (D194) analysis shows similar pattern to D90 results, hFIX-containing vectors functioned properly throughout the study. *N* = 2–5. ANOVA *p* values reported for both (d) and (e).

HemB mouse livers and spleens were collected at sacrifice or endpoint and weighed. Livers of the HemB mice trended toward significantly different, with TTR-hFIX, CB-hFIX, and TTR-PolyA averaging 1.3–1.6 g. Four mice were moribund (red dots) and had to be sacrificed prior to endpoint. They belonged to the CB (1 mouse) and CB-PolyA (3 mice) groups, and these mice had substantially lighter livers upon organ collection (Figure 6(a)). Upon organ harvest, no observable lesions or diseased areas were noticeable in the livers of the HemB mice.

The spleens of these mice, however, yielded more interesting results. Spleens from all rAAV groups were heavier than the control mice, with TTR-hFIX mice having the least heavy spleens across the rAAV groups (Figure 6(b)). The other four groups (CB, CB-PolyA, CB-hFIX, and TTR-PolyA) had at least one mouse with visible splenomegaly upon sacrifice, indicated also by weights over 0.100 g [51]. Like the C57BL/6 mice, final spleen weights do not correlate with endpoint body weight, but final liver weights correlate positively with body weights (Supplemental Fig. 5A, 5B). Interestingly, liver : spleen weight ratios showed a lower ratio for the rAAV8s encoding incomplete genomes compared to the TTR-hFIX group (Figure 6(c)). A similar ratio between the control and TTR-hFIX groups was observed, and CB, as well as CB-PolyA, ratios were significantly lower compared to that of TTR-hFIX. These results suggest that TTR-hFIX caused a minimal effect on the spleen compared to the other rAAV8 groups. To analyze the activity of the rAAV8 vectors, we ran APTT assays using plasma collected from HemB mice at day 90 and endpoint (day 194 postinjection). The clot times of TTR-hFIX injected HemB mice were significantly lower at both timepoints when compared to all other groups (Figures 6(d) and 6(e)). Of note, CB-hFIX-injected mice also exhibit a low clot time, though not to the extent of the TTR-hFIX group.

### 3.8. Ultrasound Imaging and Trichrome Staining of Livers Reveal No Differences in Echogenicity or Collagen Area of Hemophilia B Mice Injected with Various rAAV8s.

Given the published reports of HCC and liver pathologies in many mouse models of disease, we elected to image livers of the HemB mice via ultrasound to detect any possible liver masses during the study. Whole liver imaging at day 90 postinjection revealed no significant difference in echogenicity among the groups, although the TTR-hFIX group had the highest echogenicity signal. By endpoint analysis, the average pixels of each group were comparable, suggesting no detectable pathology caused by the rAAV8 at these timepoints via this modality (Supplemental Fig. 6A, 6B, 6C). A similar result was observed with the ROI near the portal triad at the endpoint analysis (Supplemental Fig. 6D, 6E). The day 90 analysis trended toward a significant difference, with the TTR-hFIX mice exhibiting the highest average pixel value compared to all other groups.

**Figure 7:**
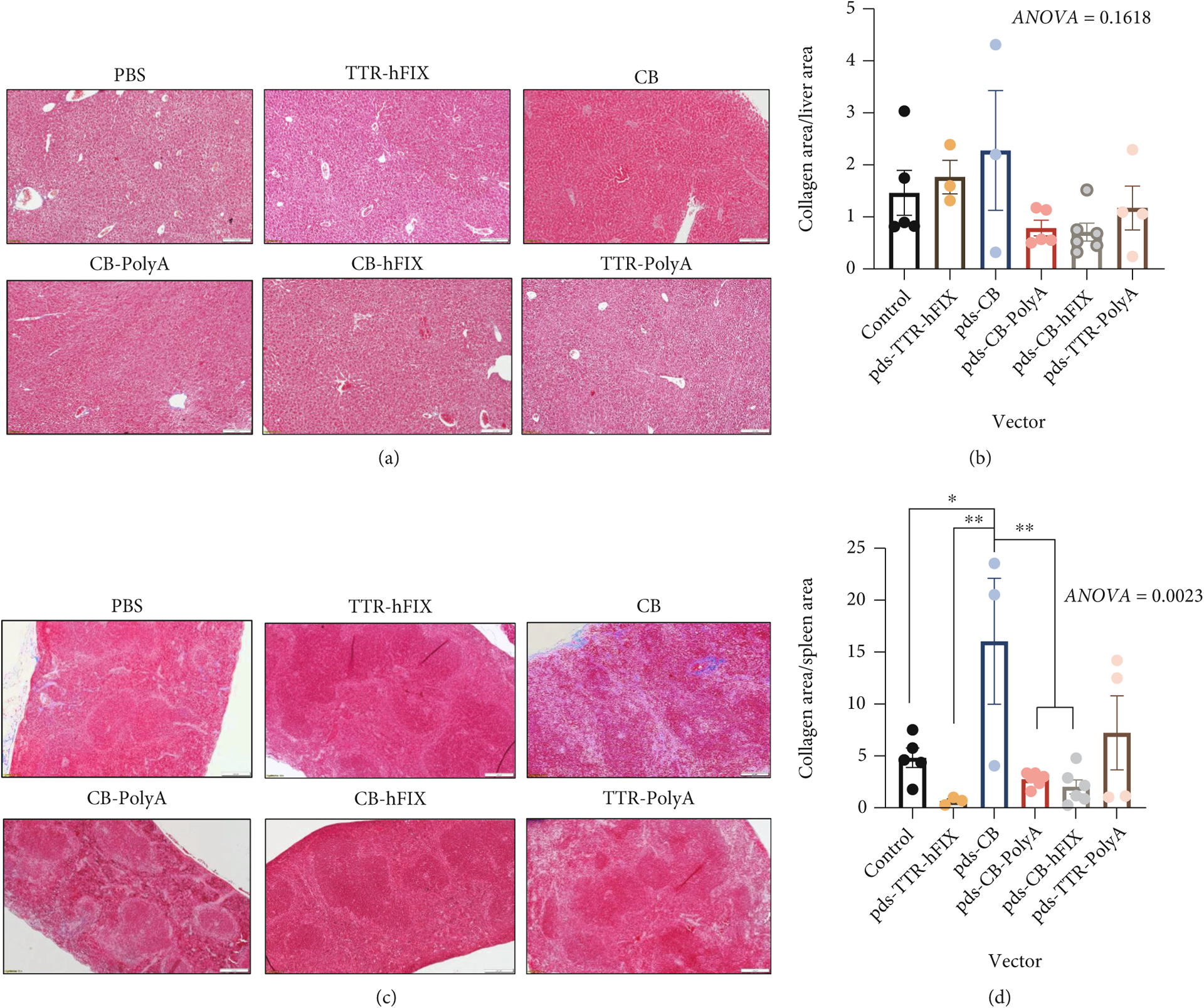
Trichrome staining of HemB mouse livers and spleens reveals differences in collagenous area of the spleens AAV-injected HemB mice. (a) Representative 10x images of collagenous area in HemB livers. (b) Collagen area analysis via microscopy. *N* = 3–6, ANOVA test performed (*p* = 0.1618). (c) Representative 10x images of collagenous area in HemB spleens. d) Collagen area analysis via microscopy. *N* = 3–6, ANOVA test performed.

In support of these ultrasound results, Masson’s Trichrome staining revealed that collagen areas of the livers were not significantly different in our HemB mice regardless of treatment (Figures 7(a), 7(b)). Our data for the HemB mice, as well as the C57BL/6 study, show a strong positive correlation between these two assessments. Moreover, ultrasound imaging seems to be a reliable noninvasive method of monitoring liver health in hemophilia B mice.

### 3.9. Trichrome Staining of Hemophilia B Spleens Shows High Collagenous Area in Mice Injected with CB and TTR-PolyA Vectors and Minimal Collagenous Area in TTR-hFIX-Injected Mice.

While we detected no differences in collagenous area in the livers, there were significant differences in collagenous area of the spleens of the HemB mice (Figures 7(c) and 7(d)). Control mice presented with approximately 5% collagen area in their spleens. Encouragingly, TTR-hFIX mice exhibited the least collagenous area, even lower than the controls, at endpoint of all groups and no splenomegaly, suggesting this rAAV8 vector did not cause a detrimental collagen deposition response in these mice and was safe. CB-PolyA- and CB-hFIX-injected mice presented with a collagen area higher than TTR-hFIX mice but lower than the control mice. TTR-PolyA- and CB-injected mice, however, had average collagenous areas greater than that of the controls, with CB mice collagen area being statistically different from those of TTR-hFIX, control, CB-PolyA, and CB-hFIX groups.

### 3.10. Pathology Report of Hemophilia B Mice Supports Safety of pDS-TTR-hFIX Vector.

**Figure 8:**
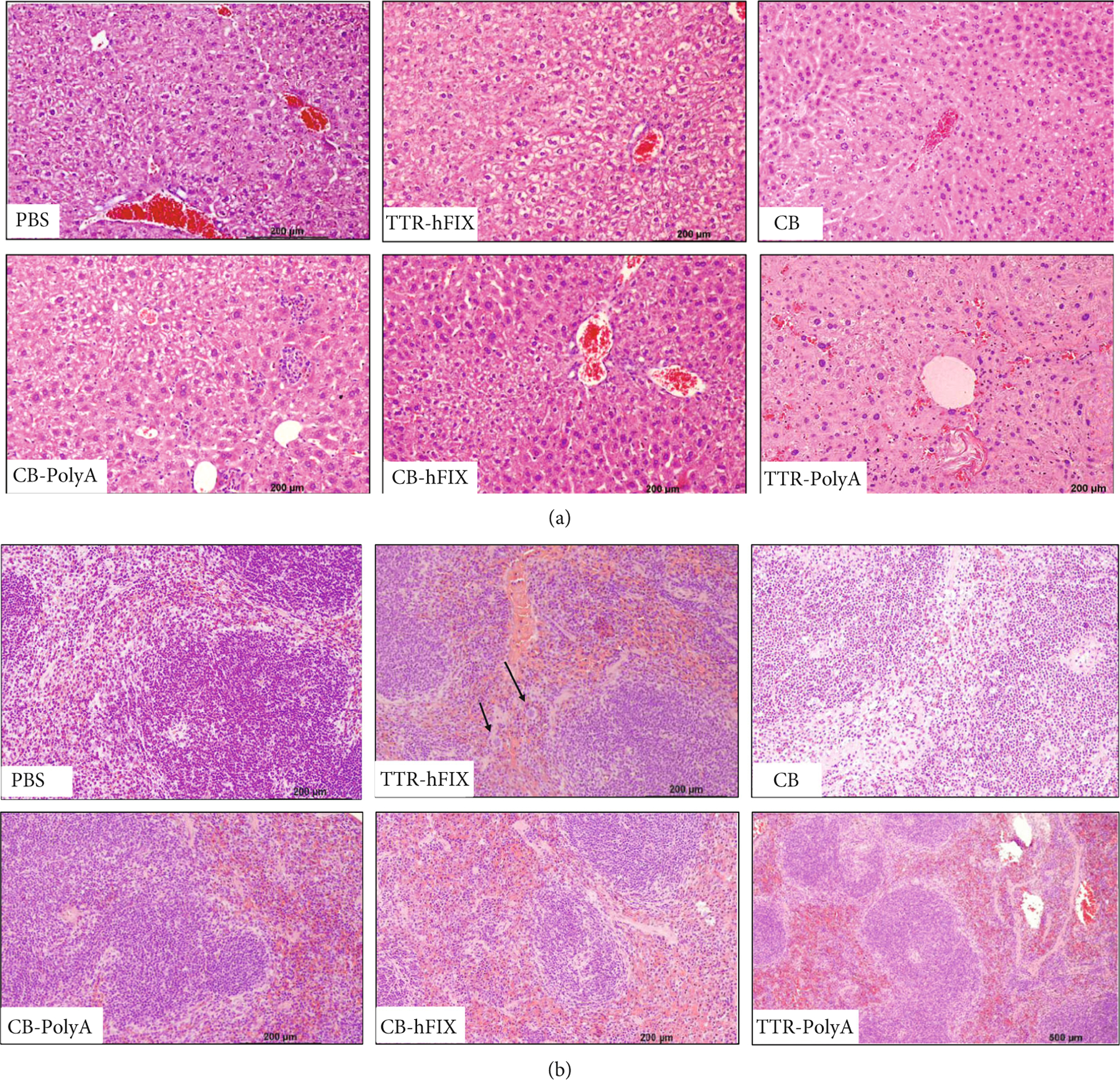

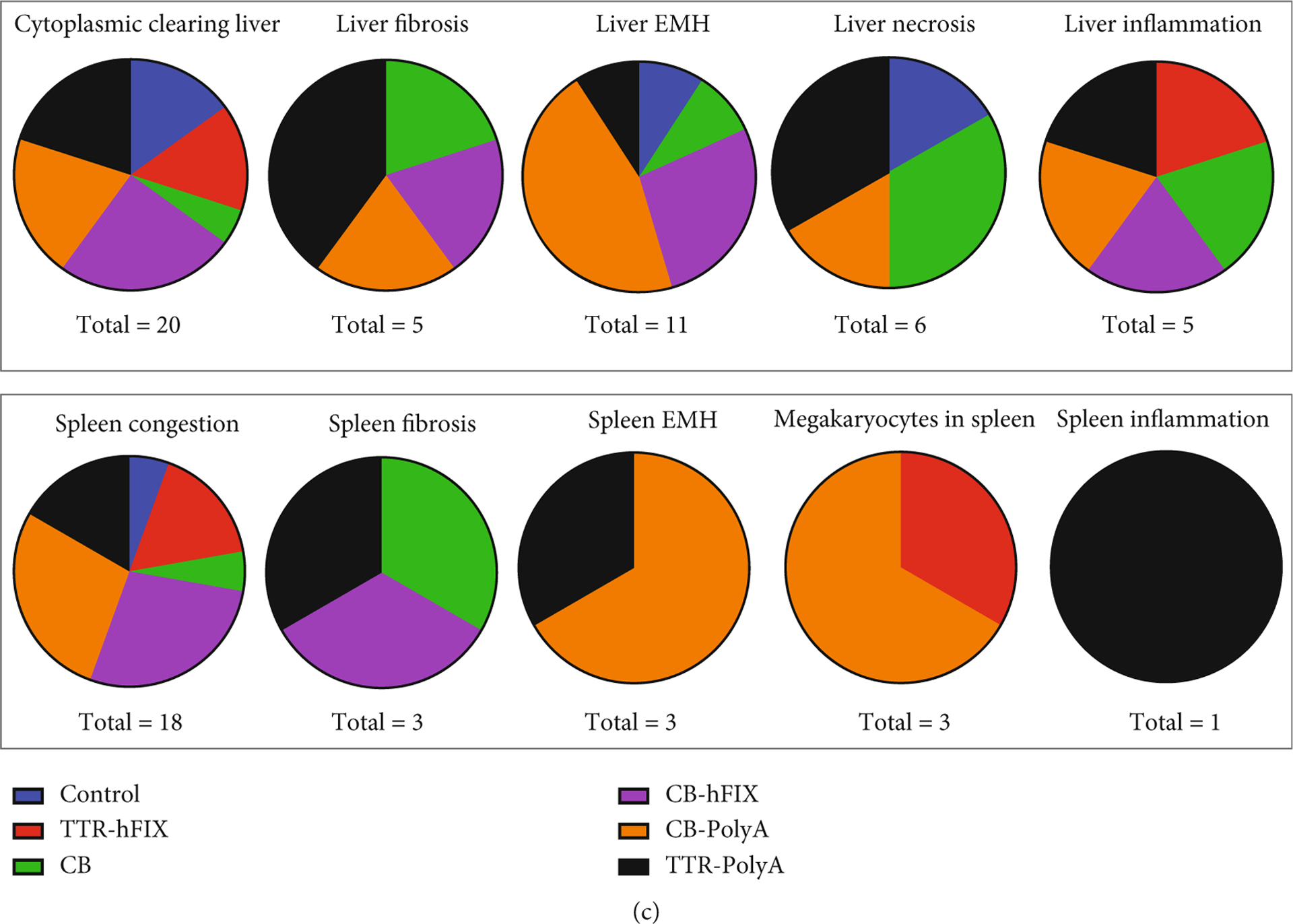
Pathology report of HemB livers and spleens via H&E. (a) Images of livers from designated groups. Cytoplasmic clearing is seen in most groups, as well as EMH. Liver fibrosis seen in TTR-PolyA. (b) Images of spleens from designated groups. Splenic congestion of red pulp was common across groups. Black arrows indicate megakaryocytes in TTR-hFIX mouse. (c) Pie charts indicating number of mice from each group positive for specific features. Most common features across groups were cytoplasmic clearing in livers and splenic congestion. Total mice analyzed for study is 28 (Control = 6, TTR-hFIX = 3, *CB* = 3, CB-hFIX = 5, CB-PolyA = 7, and TTR-PolyA = 4).

To further explore the cellularity and tissue composition of the livers and spleens of the HemB mice injected with different rAAVs, organ sections were stained with H&E and analyzed for steatosis, fibrosis, inflammation, injury, and abnormalities related to cell population, tissue structures, or growths. Across all groups, including controls, the most common feature detected in HemB livers was cytoplasmic clearing, a reversible phenomenon connected to glycogen accumulation, early cell swelling, metabolic dysregulation, and liver injury (Figures 8(a) and 8(c), Supplemental Fig. 7A) [49]. We also observed small foci of EMH in the livers of 11 mice, which can occur in response to changes in the hematopoietic environment, and was observed in some of the C57 mice; none of the HemB mice that presented with EMH were injected with the TTR-hFIX vector [52]. Liver fibrosis, indicated by collagen fibers and deposition by fibroblasts in the periportal region, was detected in 5 mice, and again, not in mice injected with the TTR-hFIX vector. These results infer that the TTR-hFIX vector had minimal negative effects on the livers of HemB mice compared to the CB-containing vectors and the TTR-PolyA vector at our 6.5-month timepoint. Importantly, no hepatocellular tumors were observed through the H&E staining, a result that matches our ultrasound imaging and Trichrome staining analyses and our data from the C57BL/6 experiment.

Evaluation of the spleens of the HemB mice revealed that congestion of the red pulp was frequently identified across all groups and typically associated with distension of the sinuses. Three mice, one each injected with the CB, CB-hFIX, or TTR-PolyA vector, had noticeable splenic fibrosis. Interestingly, megakaryocytes, immune cells involved in the production of platelets used in the initial phases of clot formation, were observed in 1 TTR-hFIX mouse and 2 CB-PolyA mice (Figures 8(b) and 8(c), Supplemental Fig. 7B). Additionally, mononuclear cells were detected in livers and spleens of several HemB mice injected with an rAAV (Supplemental Fig. 7).

To further investigate this presence on mononuclear cells, we performed F4/80 staining for macrophages in livers and spleens. We detected a significant increase in F4/80+ area in the livers CB- and CB-PolyA-injected HemB mice compared to TTR-hFIX-injected mice (Supplemental Fig. 8A, 8B). Control mice exhibited a low F4/80+ area, while the CB-hFIX and TTR-PolyA groups had moderate F4/80+ areas. Interestingly, we calculated no differences in F4/80+ area in spleens; we did notice, however, F4/80+ groups of cells situated in the marginal zones of the spleen in some mice of the CB, CB-PolyA, and TTR-PolyA groups, an area of the spleen typically negative for this marker (Supplemental Fig. 8C, 8D). Of note, one mouse from the TTR-PolyA group presented with a hemangiosarcoma in his spleen and granulomas in his liver. These pathology results, along with the Trichrome and F4/80 staining analyses, allude to potential effects of strong promoters, presence of PolyA sequences, and vectors that lack a gene target for transcription being problematic regarding spleen health, liver composition, and immune responses across organs [24]. They also demonstrate that a high dose of the completely designed TTR-hFIX rAAV8 vector caused minimal changes to liver and spleens at 6.5 months posttreatment in our HemB mouse model.

## 4. Discussion

Recombinant adeno-associated viral vectors (rAAV) are a versatile and popular gene therapy tool currently being used to study and treat myriad of genetic diseases [1–4, 7, 9, 10, 13, 18, 20, 22, 23, 40, 43, 45, 47, 53–57]. Concerns about possible links between rAAV and the development of liver cancer and fibrosis have been raised due to the occurrence of these pathologies in certain mouse models of various diseases [11–14, 21, 58]. More focus on this field of study is a key in order to determine a more comprehensive understanding of the safety and efficacy of this genetic tool and to support use of AAVs broadly in the clinic.

We explored the effects of five distinct rAAV8 vectors on liver and spleen pathology in two different mouse models, the healthy C57BL/6 line and a hemophilia B mouse line on a Balb/C background. All the C57BL/6 mice and a majority of the HemB mice lived to endpoint (day 194 post-rAAV). As groups, these mice consistently gained weight over the course of the study. The livers and spleens of rAAV-injected mice in both sets of mice weighed more than their control counterparts upon sacrifice. Collagen analysis via Masson’s Trichrome staining, H&E staining for cellularity, and ultrasound imaging to measure liver echogenicity revealed differences in liver echogenicity and collagenous areas in the liver and spleen and verified in both the liver and spleen on histopathologic analysis of certain AAV-injected mice in each of the tested mouse lines. Importantly, no liver tumors were detected via ultrasound or histology in our study, suggesting that our constructed AAVs are not directly related to HCC development in these mouse models at a timepoint of 6.5 months [59]. This data supports a broad collection of research from murine and large animal studies of various diseases, including hemophilia, and epidemiological studies of human cancers, all of which claim that AAVs are not tumorigenic [9, 19, 21, 23, 45, 47, 54, 60]. One group reports the presence of wild-type AAVs in human HCC samples, yet these analyses are retrospective and provide no evidence of causation of the HCCs due to the AAV genome [8, 17, 61].

In the rAAV-injected C57 mice, whole liver echogenicity at endpoint is lowest in TTR-hFIX mice. This result is closely mirrored in the liver Trichrome staining analysis, which shows that TTR-hFIX mice had collagenous area comparable to controls, and CB-PolyA-, CB-hFIX-, and TTR-PolyA-injected mice had elevated collagen area. While the spleens of the AAV-injected C57s were heavier than the controls, no collagen area differences were detected with Trichrome staining. In accordance with the Trichrome staining, H&E staining of the livers and spleens of C57BL/6 mice showed no liver fibrosis or inflammation but did reveal EMH and cytoplasmic clearing in the livers across several groups, and hemosiderin in mouse spleens also in many of the different groups. Many of the aforementioned features are related to organ injury. It is reported that use of cisplatin, a platinum-based chemotherapy, and a CXCR3 inhibitor used to reduce inflammation, caused hemosiderin accumulation in rodent models of disease [50, 62]. In connection with hemophilia, Sun et al. published data showing that hemosiderin deposition in joints decreased in a mouse model of HemA when AAV8-FVIII was supplemented with recombinant AAV9-FactorVIIa, which was used to prevent the effects of autoinhibitory antibodies [63].

In the HemB mice, liver echogenicity and collagen analysis correlated positively in showing no differences among the groups. Interestingly, collagen area in the spleens of TTR-hFIX HemB mice was lower than the controls and all other AAV-injected groups; CB-injected HemB mice exhibited the highest splenic collagen area. The pathology report of the HemB mice illuminated several detailed features of liver injury, EMH, spleen injury, and presence of specific immune cells that connected and supported data gathered from organ weights, ultrasound imaging, and Trichrome staining. Control mice demonstrated no inflammation in livers. CB-injected and TTR-PolyA-injected HemB mice had detectable fibrosis, extramedullary hematopoiesis (EMH), necrosis, and inflammation in their livers. Comparatively, TTR-hFIX-injected HemB mice presented with splenic cytoplasmic clearing (2 mice) and inflammation (1 mouse).CB-hFIX and CB-PolyA HemB mice had high levels of liver EMH as well as cytoplasmic clearing. In correlation with these results, F4/80 staining showed the TTR-hFIX HemB mice exhibited the smallest F4/80+ area in their livers compared to all other groups. F4/80 cells, which identifies Kupffer cells and macrophages, are known to contribute to fibrosis [56]. Overall, these results demonstrate ranges of possible pathological effects of rAAV design and promoters on liver and spleen health, with TTR-hFIX vector causing minimal changes in echogenicity, liver and spleen composition, and collagen deposition when compared to multiple mice injected with the vectors containing the CB promoter and the TTR-PolyA vector.

Interestingly, of the rAAV-injected mice, those given the CB vector had the greatest collagenous area in their spleens in both mouse models tested. In the spleens of HemB mice, this CB group had significantly detectable fibrosis and congestion. We recognize the relatively small number of HemB mice (*N* = 3) that received an I.V. pDS-AAV-CB rAAV8 injection as a limitation of our data. This limitation was due to restricted availability of HemB mice from our collaborator’s lab that has active research on Hemophilia B, and the loss of one CB-injected mouse at D0 due to an embolism during injection. Nevertheless, this result strengthens the importance of vector design and the use of specific promoters for specific illnesses and performing in-depth immune profiling of spleens for future AAV studies, as this organ seemed to be greatly affected in the HemB mouse model in our study.

Our results also demonstrate a strong positive correlation between the analyses used to assess liver fibrosis, the ultrasound imaging and the two histological staining protocols. This encouraging result suggests that non-invasive ultrasound imaging across AAV experiments could be used to detect gross anatomical changes in multiple soft organs early on and at multiple timepoints for long-term experiments [27, 28, 30]. Adverse events due to AAV injection could be detected early using ultrasound, which would permit better analysis of AAV effects in animal models.

These reported results support the concept that AAVs are not tumorigenic as no HCC was detected in any of our AAV-injected mice over the 6.5-month timeline. No AAV has been directly linked to cancer development; therefore, we lacked a true positive control for these studies, but our results do support the documented safety of AAVs [18–21]. While we detected differences and observed changes in spleen and liver tissue in our mouse models, the selected timeline of 6.5 months is in the short to moderate range compared to other studies using AAV [7, 9, 11–14, 18, 21, 24, 38, 47, 64]. Hemangiosarcomas can arise spontaneously in older mice, and hepatic microgranulomas are often idiopathic and an incidental finding in aging mice. Yet, our mice lived to be 8.5 months old, which is considered near middle-aged for this animal model. While not found in the spleen, a research group reported development of hemanigosarcomas in fat tissue of C57BL/6 mice at 4.5 months of age injected with a lung-targeted AAV6 vector encoding the envelope protein of the Jaagsiekte sheep retrovirus, suggesting that presence of this type of tumor could be related to contents of AAV [65]. Longer term studies of at least 1 year and a range of rAAV doses using these same vectors and greater numbers of mice in each group could reveal greater differences in collagen area and potential fibrosis or other pathologies associated with the liver and spleen.

Additionally, testing vectors with other commonly used strong promoters besides CB and TTR, such as elongation factor 1 alpha (Ef1*α*), thyroxine binding globulin (TBG), and cytomegalovirus (CMV), could reveal details about how promoters may be specifically contributing to changes in liver and spleen composition. Moreover, staining the livers and spleens for specific immune cells and inflammatory proteins would enlighten about mechanisms regarding splenomegaly and collagen deposition we observed in our studies [42, 43]. The pathology reports indicate the presence of megakaryocytes (MKs) and mononuclear populations in the livers and spleens of some mice. We confirmed the mononuclear result via F4/80 staining, detecting greater F4/80 area in CB and CB-PolyA HemB livers compared to TTR-hFIX HemB mouse livers, and potential spatial disruptions of this cell population in CB, CB-PolyA, and TTR-PolyA HemB spleens. It is reported that MKs and monocyte-derived macrophages, along with various types of endothelia, can produce clotting factor proteins [56, 66]. Specific immunohistochemistry for a wide variety of immune subpopulations, particularly macrophages and other mononuclear cells, and colocalization of Factor IX in the HemB mouse groups would provide quantifiable analysis of any potential differences in immune responses caused by these different AAVs and whether any relationship exists between the type of injected rAAV, and immune cell infiltrates found in livers and spleens.

We report that the liver weights and final mouse body weights correlated, underscoring the need for more comprehensive and specific analyses beyond weight as an indicator of pathology. The liver is the largest internal organ in mice and accounts for approximately 3–5% of the animal’s body weight [35]. Notably, the area of collagen in each liver did not correlate with weight (data not shown). The addition of biochemical assays to assess liver enzymes such as aspartate transaminase (AST) and alanine aminotransferase (ALT) would be a strong supplement to the ultrasound imaging, pathology analysis, and the Trichrome staining we present here in this manuscript [48].

A well-documented mechanism of HCC development reported in various mouse models treated with AAVs is genomic integration of the vector in manners that activate oncogenes or silence tumor suppressors [12–14, 58, 64]. Strong promoters, such as CB and CMV, are reported to exhibit read-through activity, a mechanism that is problematic in causing genomic integration of AAV and activation of off-target genes [12–14, 64]. Donsante et al. show that a sequence of the 5′UTR of the AAV2 vector encoding GUSB was discovered in murine cancerous liver tissue of MPSVII mice analyzed 9–18 months postinjection; this AAV2 contains the *β*-actin promoter (CBA) and a CMV enhancer, both considered strong *cis* AAV elements [12]. Chandler et al. published data that also show the CBA promoter; this time in an AAV8 vector, was linked to increased HCC occurrence in a mouse model of methylmalonic acidemia (MMA). This effect was also dose-dependent and seen after 22 months of treatment. Interestingly, the same effect was not seen in MMA mice injected with an AAV8-MUT vector that housed the *α*−1 anti-trypsin (AAT) promoter, a weak liver-specific promoter [13]. Li and colleagues published data in support of the AAT promoter not inducing HCC development, even at a high dose of 1 × 10^14^ vg/kg [64]. Rosas et al. report that C3H/HeJ mice at 9–15 months of age injected with 2 × 10^12^ of a CB-containing null scAAVrh74 developed HCC at a greater rate than noninjected controls and mice injected with a CMV-GFP scAAVrh74; this tumor development was exacerbated when coupled with liver injury. The same effect was not seen in SCID mice injected with these same scAAVs and liver injury regimens [14]. Results such as this reinforce how promoter choice, vector dose, mouse strain and disease model, and vector design can influence organ health and cause different side effects from AAVs in vivo.

Future work assessing any potential AAV components that integrated into the livers and spleens could provide insight into which components of these vectors could be important for preventing or facilitating integration. Prior work from our group has revealed the production of satellite subgenomic particles during the AAV life cycle in cell culture models that could be problematic in leading to genomic integration and a possible cancer risk [10, 39, 67]. One class of subgenomic particles, snapback genomes (SBGs), can contain promoter-only genomes, a vector that could be risky for genomic integration and read-through if insertion occurs in an oncogene. Indeed, the mice injected with the pDS-AAV-CB vector, which can mimic a strong promoter-only SBG, presented with elevated collagen deposition in the spleens, high F4/80+ area in livers, and several liver pathologies in the case of the HemB cohort. The vectors used in this paper should be analyzed for genomic integration and production of different SBGs that are created, as one would expect them to be unique based on their specific promoter and the presence of a Poly-A sequence and the gene of interest.

Of note, 3 HemB mice in the CB-PolyA group that were moribund presented without spleens upon sacrifice. Deficiencies in plasminogen are reportedly linked to cases of splenic rupture, but no such rupture has been linked to hemophilia B [68]. Isolation of primary cells from hemophilic spleens and conducting in vitro studies with different AAV vectors could reveal the reason for why these mouse spleens were not present upon sacrifice.

## 5. Conclusion

Overall, our results reported herein demonstrate that rAAV8 vectors containing either the liver-specific TTR promoter or the strong CB promoter that either contain or lack a PolyA sequence and the transgene encoding human FIX have distinct effects on liver and spleen health yet caused no HCC development during the 6.5-month experiments in the inbred C57BL/6 or the Balb/C hemophilia B mouse models. We analyzed the animals, livers, and spleens by mouse and organ weights, liver echogenicity, Masson’s Trichrome and H&E staining, F4/80 immunohistochemistry, and veterinary pathology reports. Ultrasound imaging of the livers, a noninvasive imaging modality used during the in vivo studies, is sensitive enough to detect composition differences that correlated well with histopathology. Veterinary pathology reports identified several biological features and cellular composition details that should be investigated further. More detailed analysis of rAAV design and how specific DNA components affect organ health will lead to greater efficacy, safer design, and broader use of AAVs in the clinic.

## Supplementary Material

supplementary Material

## Data Availability

The in vivo and in vitro data used to support the findings of this study are available from the corresponding author, Dr. Weidong Xiao, upon request.
