# Supplementary material for: Genomic Designs of rAAVs Contribute to Pathological Changes in the Livers and Spleens of Mice
Source: Adv Cell Gene Ther. Author manuscript; Available in PMC 2022 Dec 8. (PMC9730939; doi:10.1155/2022/6807904)
Supplement: supplementary Material [file NIHMS1853850-supplement-supplementary_Material.docx]

**Genomic Designs of rAAVs Contribute to Pathological Changes in Livers and Spleens of Mice:**

**Supplemental Figures, Table, and Figure Legends**

**
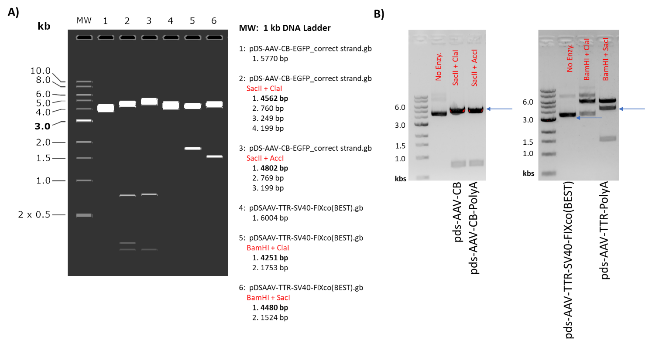
**

**
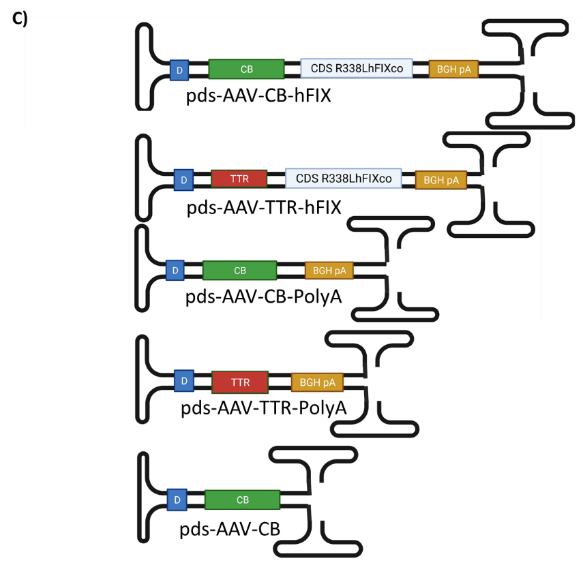
**

**Supplemental Figure 1**: Gel Preparation of Vectors for In Vivo Studies: A) *SnapGene* Gel Simulation. Lane assignments, restriction enzymes, and expected band sizes are listed. B) Actual 0.8% agarose gels. Blue arrows indicate bands of vectors used for *in vivo* studies. C) pDS-AAV8 genomes and names. Black end structures are ITR (5’) and mutant ITR (3’). Created with BioRender.com ITR= Inverted Terminal Repeat Sequence, CB= Chicken Beta Actin, TTR= Transthyretin, D= D Sequence, BGH pA= Bovine Growth Hormone polyadenylation signal.

**
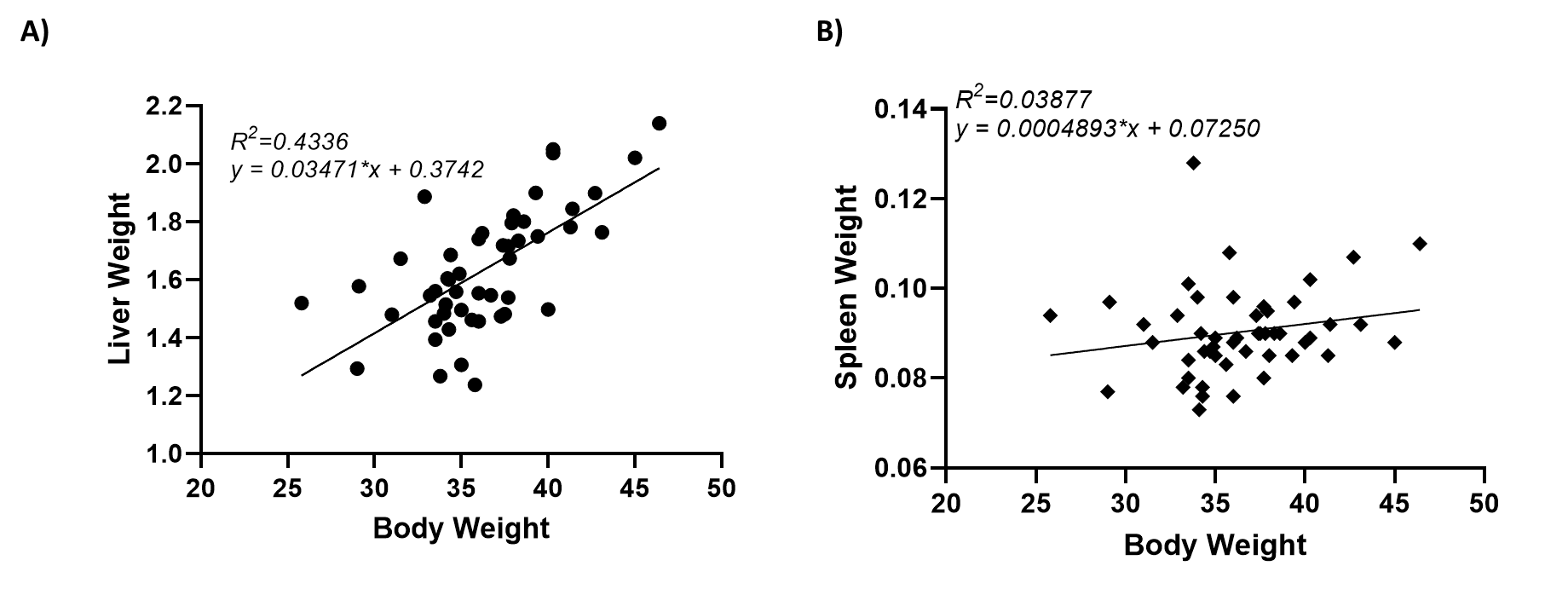
**

**Supplemental Figure 2**: Liver Weights, but not Spleen Weights, Correlate with C57 Bl/6 Mouse Endpoint Weights. A) Liver weights vs Endpoint Body weight analyzed by Pearson’s correlation; p<0.0001, N=49. B) Spleen weights vs Endpoint Body weights analyzed by Pearson’s correlation; p=0.1745, N=49.

**
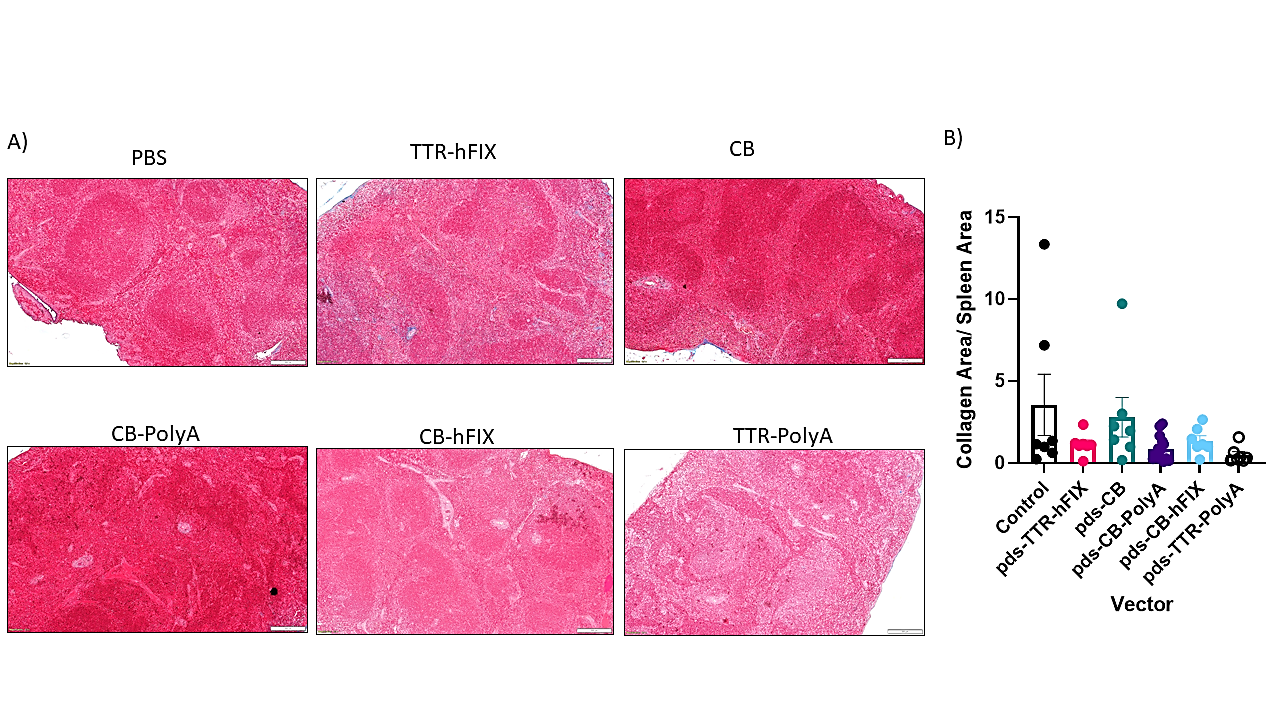
**

**
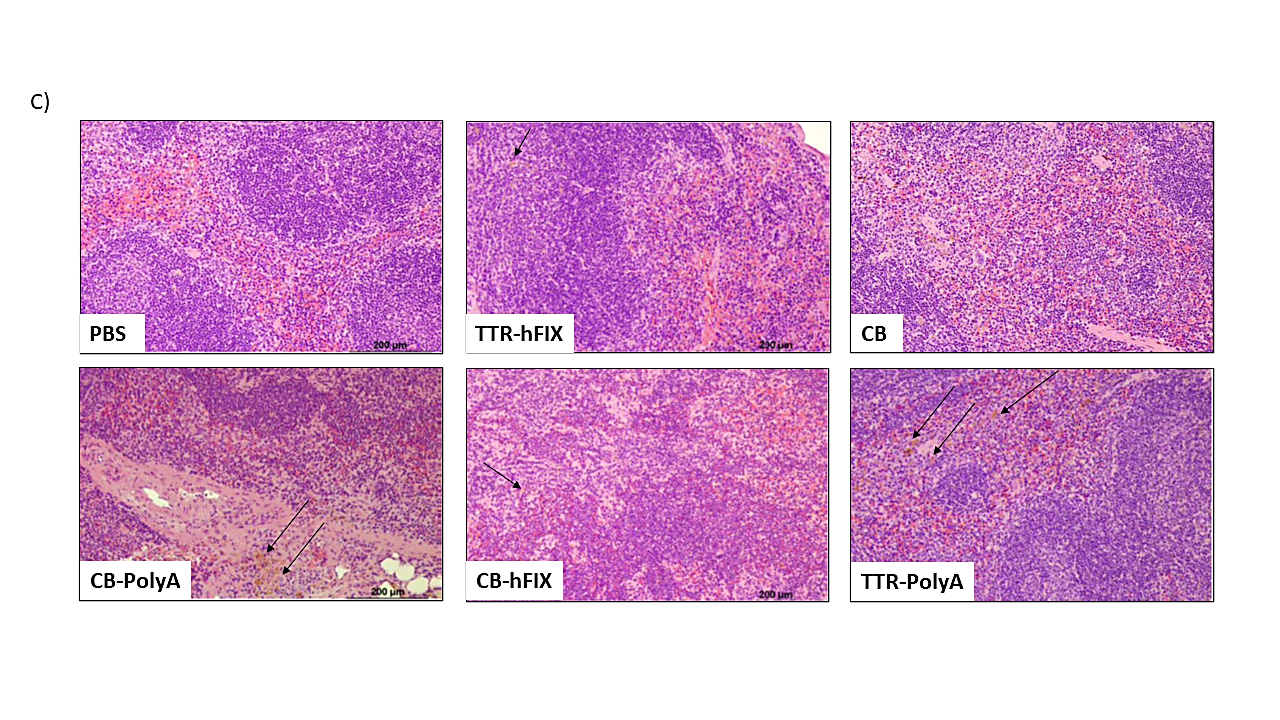
**

**Supplemental Figure 3:** Masson’s Trichrome and H&E Stains of C57/Bl6 Spleens. A) Representative 10x Images of Collagenous Area in C57 Spleens. B) Collagen area low in Spleens of TTR-hFIX, and high in CB Injected Mice. Kruskill-Wallace test performed. *p=0.1106.* C) H&E 20x images of C57 Spleens. Black arrows indicate hemosiderin deposits found at mild levels in several vector-injected mice across groups.

**
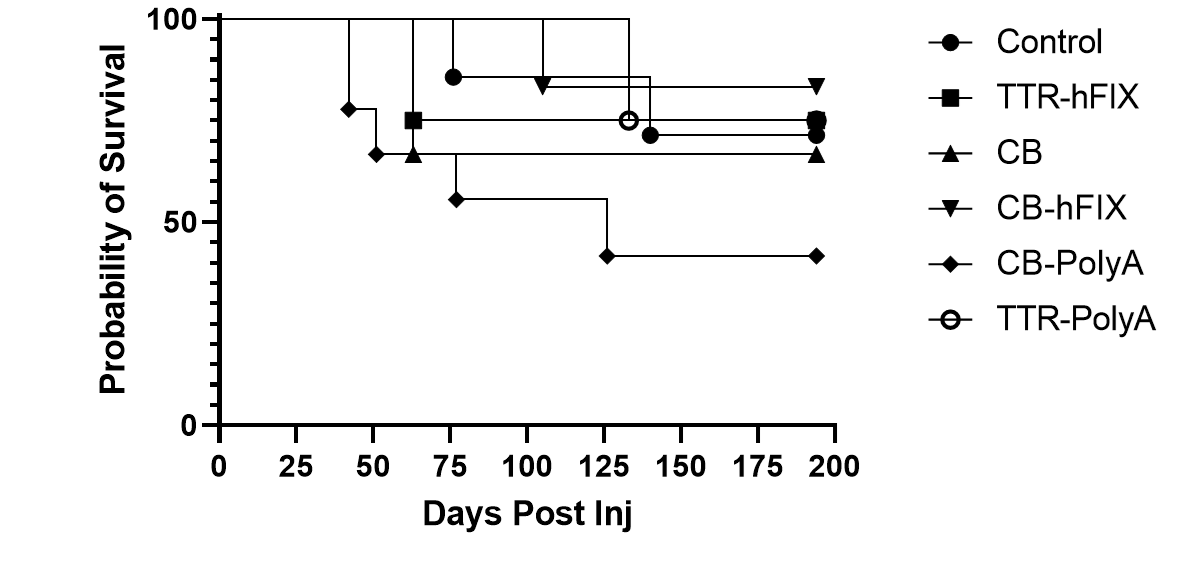
**

**Supplemental Figure 4:** Survival Graph of Hemophilia B Mice Groups. Kaplan-Meier curve demonstrates no difference in survival among groups. N=3-9*. p=0.4331*

**
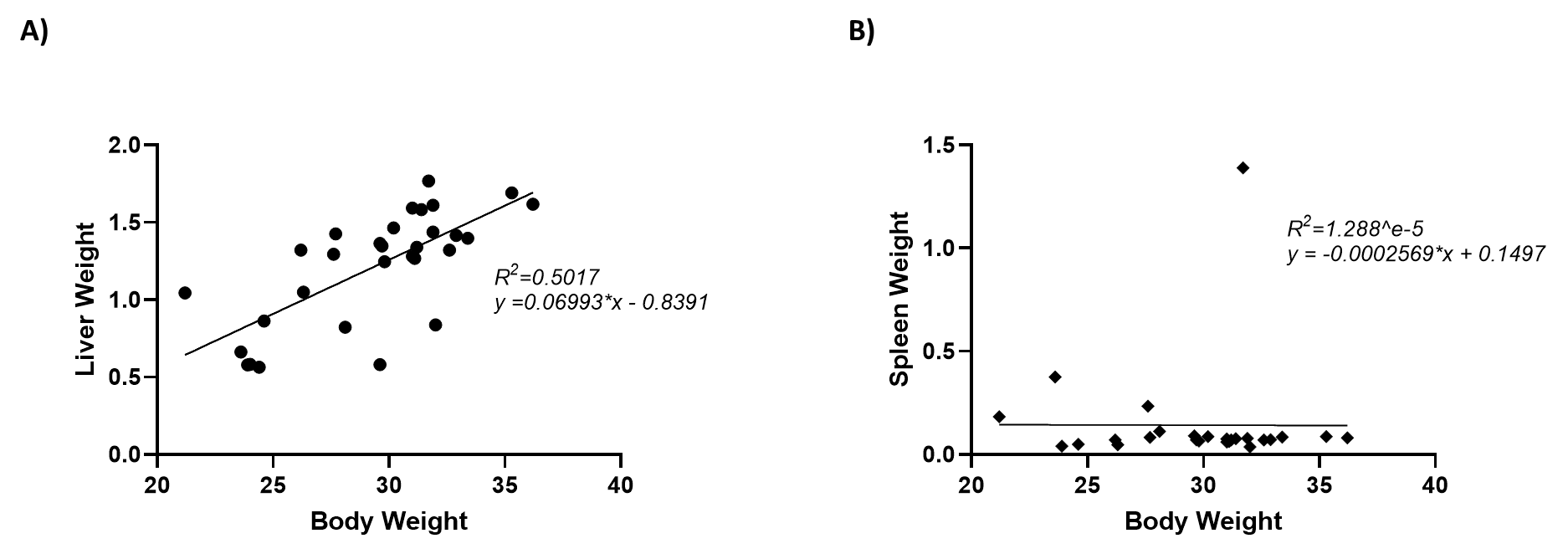
**

**Supplemental Figure 5:** Liver Weights, but not Spleen Weights, Correlate with Hemophilia B Mouse Endpoint Weights. A) Liver weights vs Endpoint Body weight analyzed by Pearson’s correlation; p<0.0001, N=29. B) Spleen weights vs Endpoint Body weights analyzed by Pearson’s correlation; p=0.9858, N=27.

**
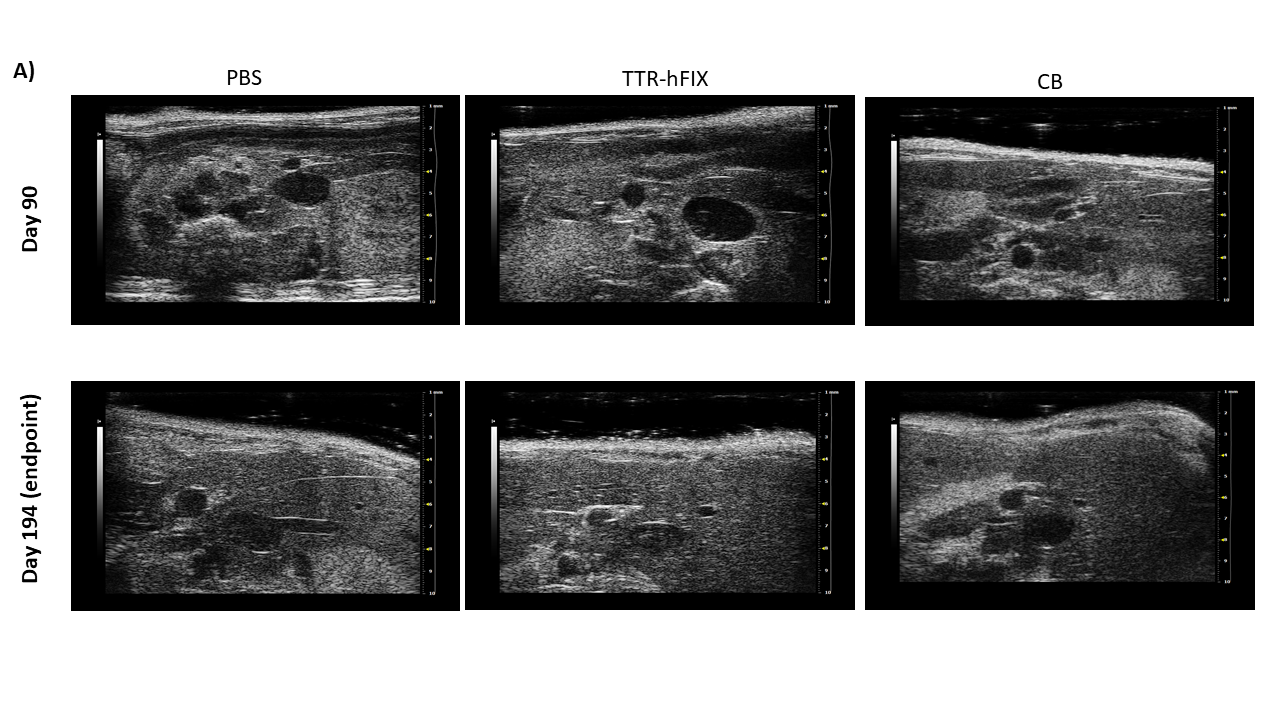
**

**
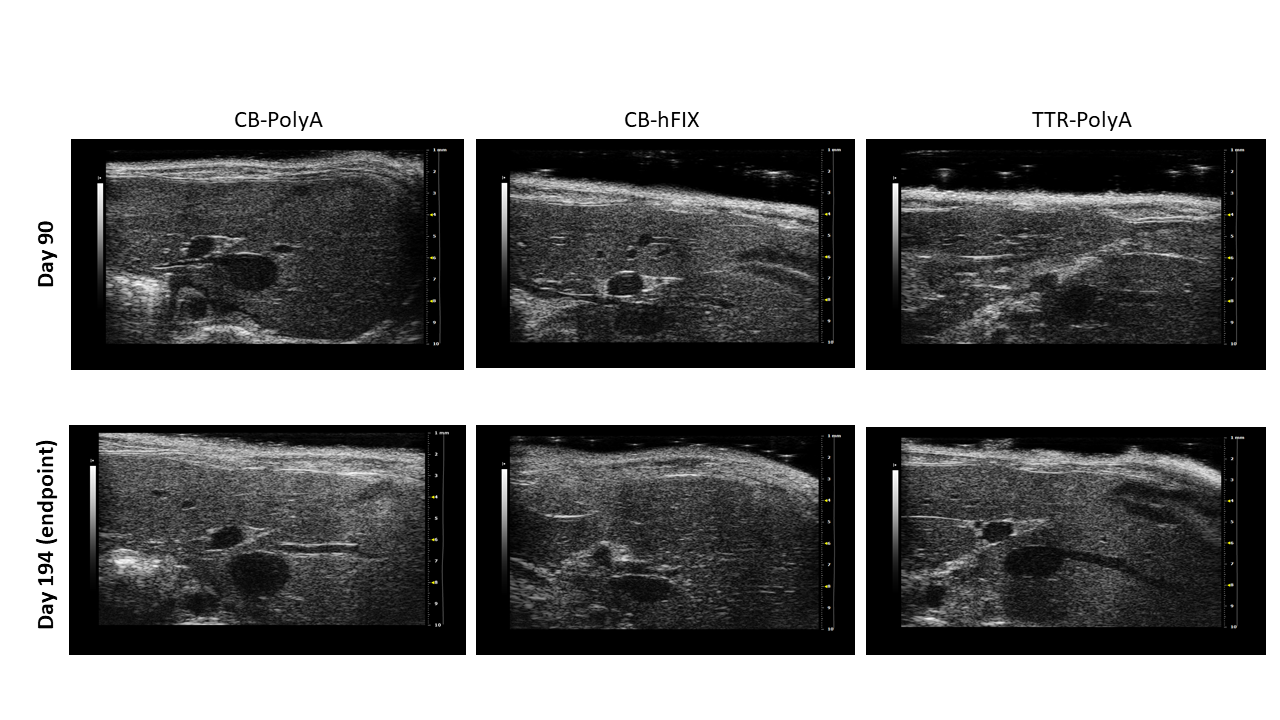
**


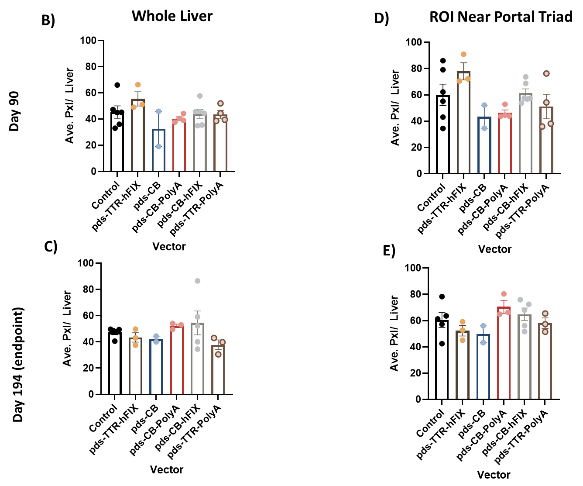


***p=0.2031***

***p=0.3957***

***p=0.0913***

***p=0.2017***

**Supplemental Figure 6:** Echogenicity measurement of all Hemophilia B mice injected with AAV. A) Representative Day 90 and Day194 liver ultrasound images for all groups of HemB Mice. Arrows indicate Portal Triad features on D90 images: Blue- Portal Vein, Red- Hepatic Artery, Green- Bile Duct. B) & C) Whole Liver Echogenicity analysis via average pixels of liver images. N=2-6. D) & E) Echogenicity analysis of a region of interest near the Portal Triad. Kruskill-Wallace test performed for B, and D (*p= 0.2017 & p=0.0913*). ANOVA performed for C and E (*p= 0.3957, & p= 0.2031*).


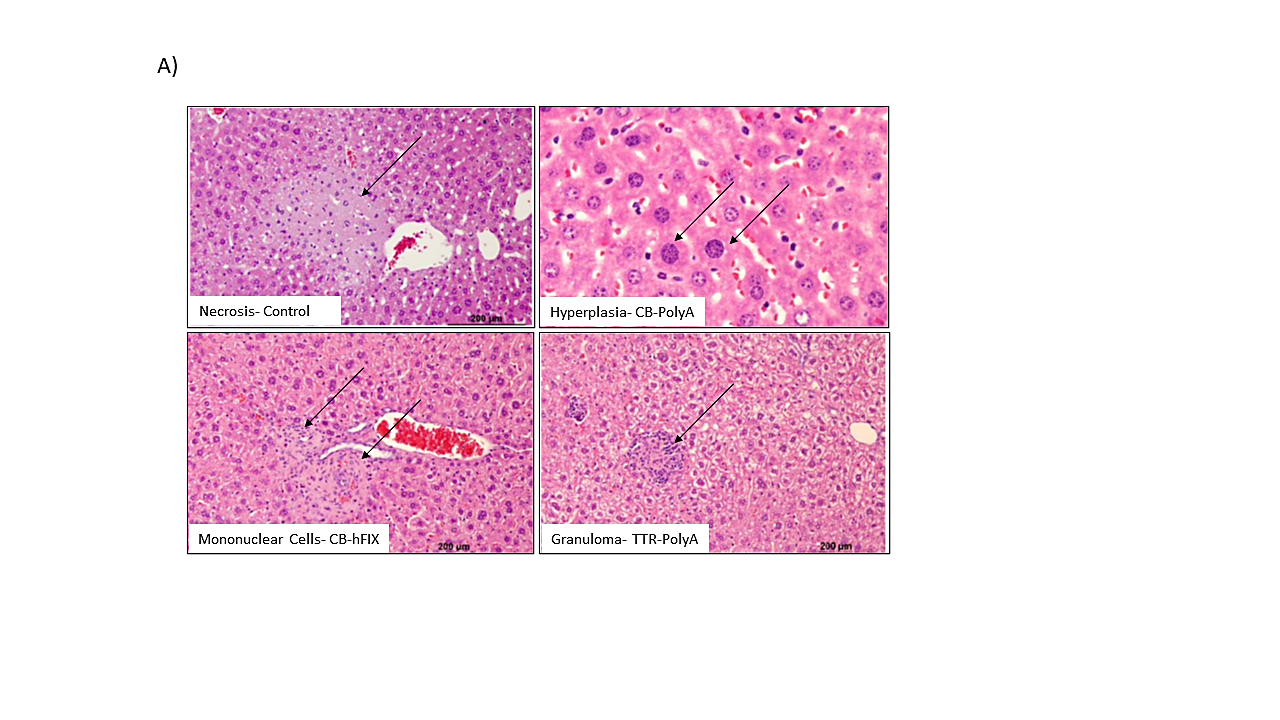

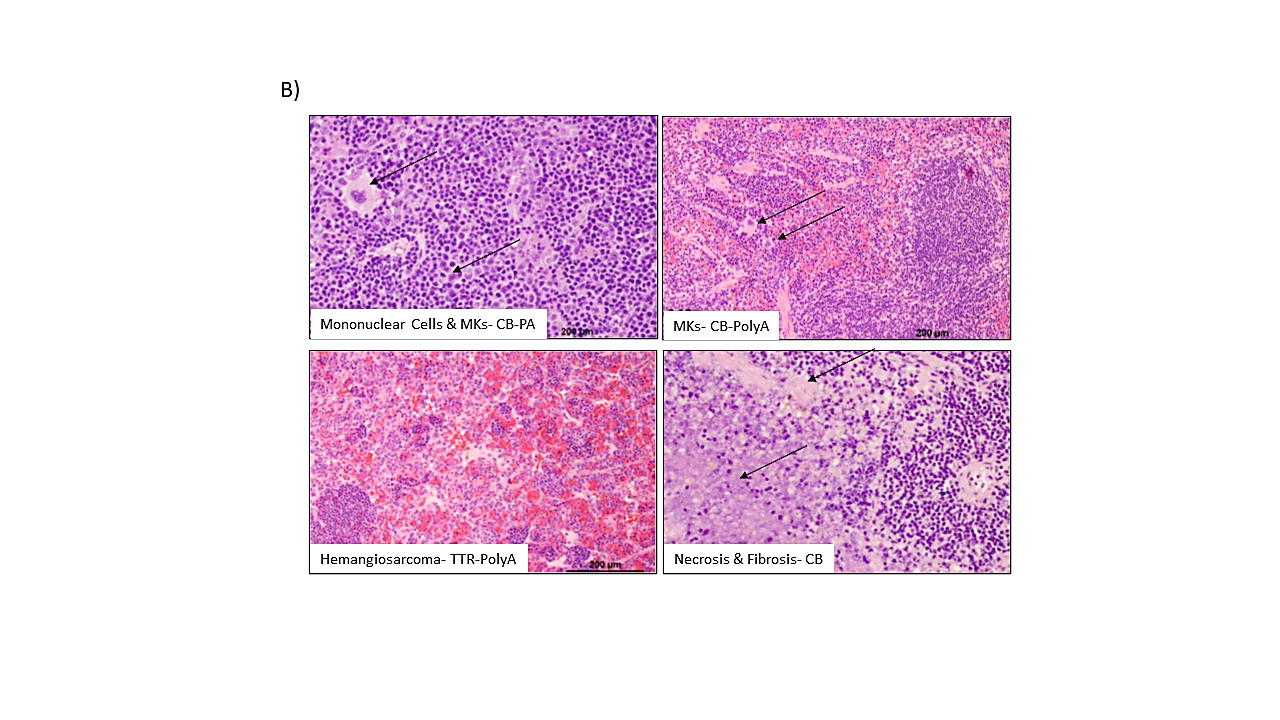


**Supplemental Figure 7:** Specific Histopathological Features of AAV-injected HemB Mice. A) Liver features detected in various mice via 20x and 40x images. Black arrows indicate listed feature. B) Spleen features detected in mice via 20x and 40x images. Black arrows identify features. The hemangiosarcoma is found throughout image.


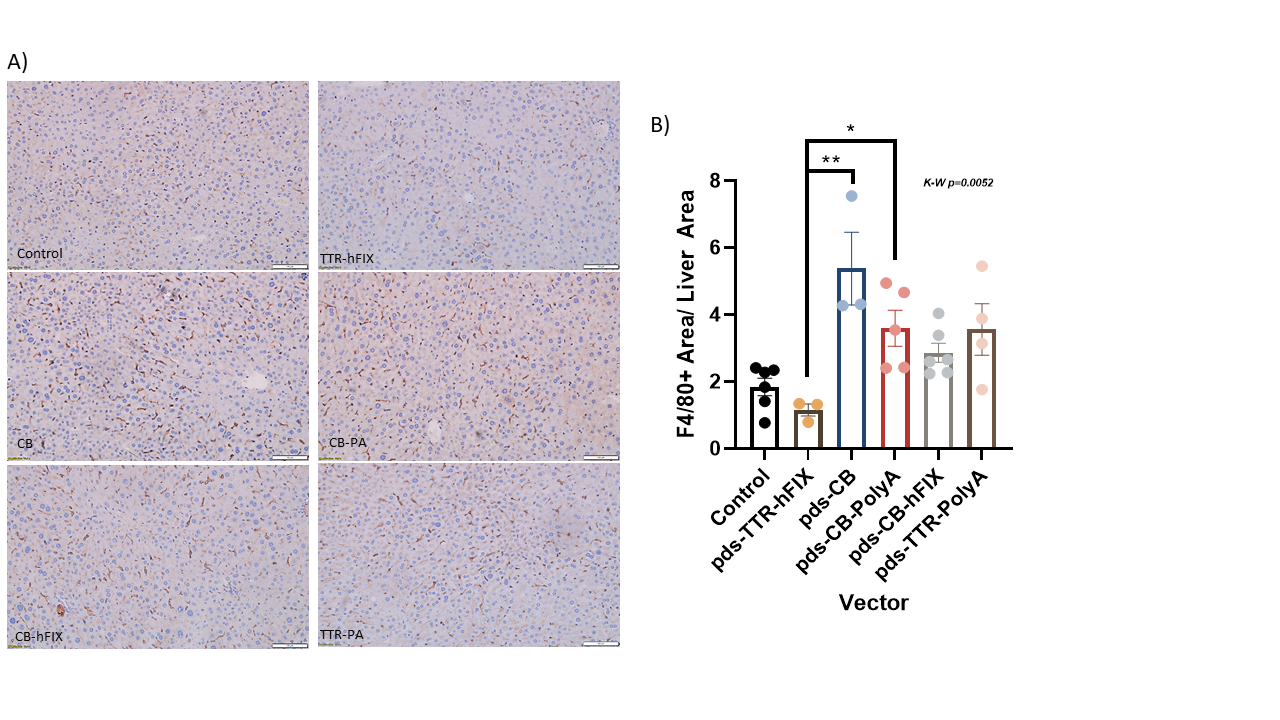


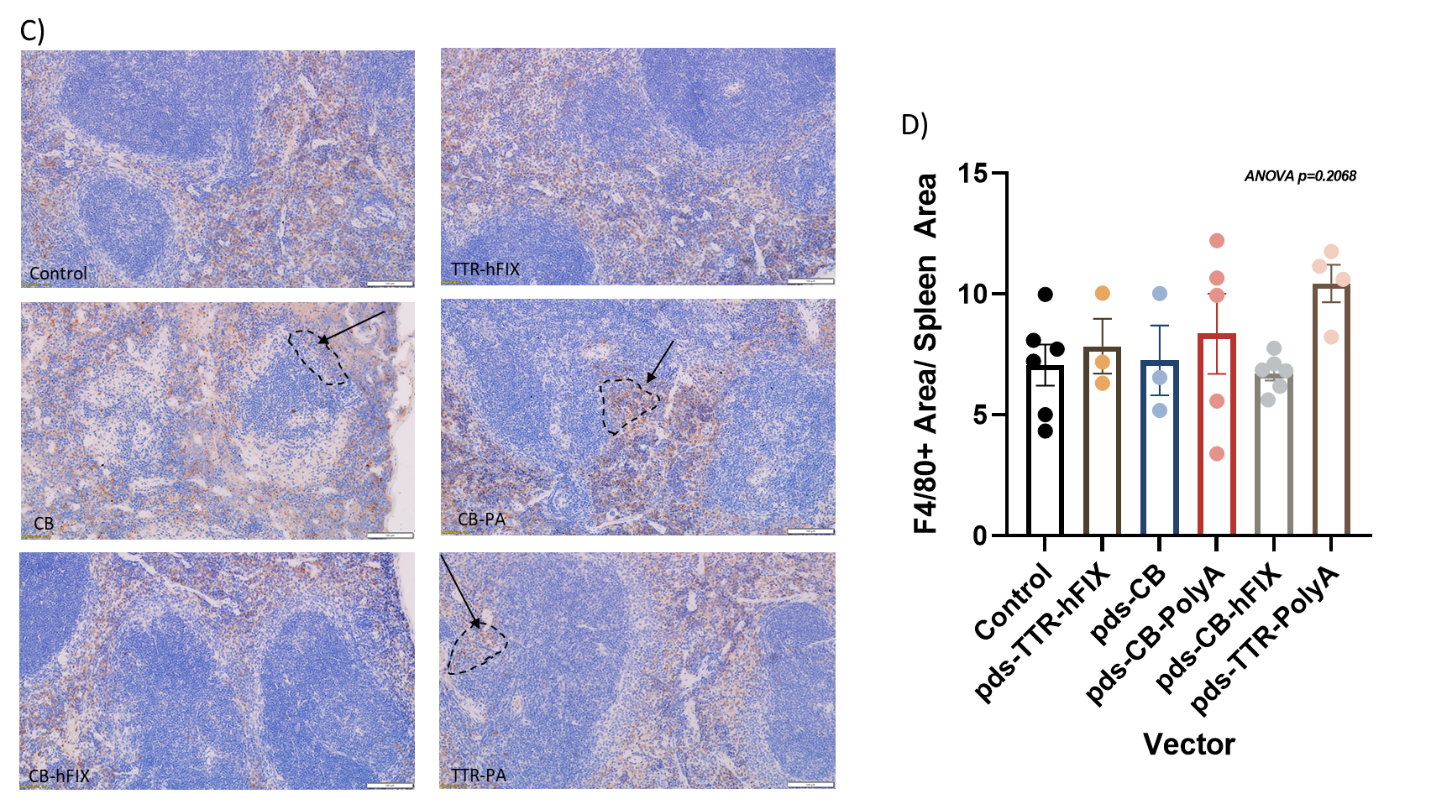


**Supplemental Figure 8:** HemB Mice Injected with rAAVs Containing Incomplete Genomes Have Elevated F4/80+ Area in Livers but not in Spleens. A) 20X liver images. Kupffer cells and macrophages detected via F4/80. B) Quantification of F4/80+ area in liver. TTR-hFIX contain significantly lower % of cells compared to CB and CB-PolyA groups via Kruskill-Wallace test. C) 20X spleen images. Red pulp macrophages identified with some cells detected in the marginal zones in HemBs injected with incomplete genome rAAVs. Black arrows and dotted traces indicate possible encroachment of F4/80+ macrophages from the red pulp to marginal zones and white pulp in certain groups. D) Quantification of % F4/80+ area in spleen. No significant differences among groups via One-way ANOVA.

| ***scAAV8*** | ***5’ Primer*** | ***3’ Primer*** |
| --- | --- | --- |
| scAAV8-TTR-hFIX-PolyA | TGAGTGCACTGTGTTCCTG | CAGCTTGCCAGAGTTGTATCT |
| scAAV8-CB | GTCAATAATGACGTATGTTCCCATAG | CCGTAAATACTCCACCCATTGA |
| scAAV8-CB-PolyA | GTCAATAATGACGTATGTTCCCATAG | CCGTAAATACTCCACCCATTGA |
| scAAV8-CB-hFIX-PolyA | TGAGTGCACTGTGTTCCTG | CAGCTTGCCAGAGTTGTATCT |
| scAAV8-TTR-PolyA | ACTGTGCCTTCTAGTTGCC | TAGGAAAGGACAGTGGGAGT |

**Supplemental Table 1:** scAAV8 Titer Primer Pairs.
